# Spatially coordinated cell cycle activity and motility govern bifurcation of mammary branches

**DOI:** 10.1083/jcb.202209005

**Published:** 2023-06-27

**Authors:** Satu-Marja Myllymäki, Beata Kaczyńska, Qiang Lan, Marja L. Mikkola

**Affiliations:** 1 Institute of Biotechnology, Helsinki Institute of Life Science, University of Helsinki, Helsinki, Finland

## Abstract

Branching morphogenesis is an evolutionary solution to maximize epithelial function in a compact organ. It involves successive rounds of branch elongation and branch point formation to generate a tubular network. In all organs, branch points can form by tip splitting, but it is unclear how tip cells coordinate elongation and branching. Here, we addressed these questions in the embryonic mammary gland. Live imaging revealed that tips advance by directional cell migration and elongation relies upon differential cell motility that feeds a retrograde flow of lagging cells into the trailing duct, supported by tip proliferation. Tip bifurcation involved localized repression of cell cycle and cell motility at the branch point. Cells in the nascent daughter tips remained proliferative but changed their direction to elongate new branches. We also report the fundamental importance of epithelial cell contractility for mammary branching morphogenesis. The co-localization of cell motility, non-muscle myosin II, and ERK activities at the tip front suggests coordination/cooperation between these functions.

## Teaser

This study shows that mammary gland branching morphogenesis is orchestrated by spatiotemporally coordinated changes in tip cell motility and proliferation to alternate between branch elongation and branch point formation.

## Introduction

Branching morphogenesis is a conserved developmental program that molds an epithelial tube or chord into the form of a branched tree. As a result, the functional surface area of the epithelium is increased manifold—a principle adopted by the lungs, kidneys, and many secretory organs, including the mammary gland. During development, each organ follows a unique pattern of epithelial growth and branching, orchestrated by inductive signals from the surrounding mesenchymal tissue (Lang et al., 2021). Inductive signals elicit changes in epithelial cell behaviors leading to elongation of branches and generation of new ones. New branch points can form at two topological domains of the growing epithelium. Branches can sprout from the side of an existing branch, referred to as lateral or side-branching, but this has only been documented in the lung and mammary gland (Iber and Menshykau, 2013). In all branching organs, the blunt-ended tips of branches can split into two or more branches—a process called terminal bifurcation or clefting. Between bifurcations, the tips invade into the mesenchymal tissue thereby elongating the stalk of the branch, also called “trailing duct.”

Mammary gland is a unique arborized organ as branching morphogenesis takes place in three developmental stages—embryonic, pubertal, and reproductive. In mice, branching morphogenesis begins when a sprout rooted to the embryonic surface ectoderm is induced to branch around embryonic day 16 (E16) and yields a rudimentary tree of 10–20 branches by birth (E19-20; Macias and Hinck, 2012; Spina and Cowin, 2021; Lindström et al., 2022 *Preprint*). Lumen formation commences shortly before birth, and soon after, the final, bi-layered structure of mammary ducts is established, and ductal tree continues to grow isometrically to the body size (Hogg et al., 1983). The inner layer of cells enclosing the lumen and outer layer of cells separated from the mesenchyme by a basement membrane, begin to segregate into distinct luminal and basal lineages during late embryogenesis (Lilja et al., 2018). During puberty, hormones instigate a massive epithelial expansion, characterized by invasion and bifurcation of the proliferative terminal end buds (TEB) containing multiple layers of luminal cells surrounded by a single layer of basal cells (Paine and Lewis, 2017). In the adult gland, hormonally induced side-branching occurs during the estrous cycle and pregnancy (Brisken et al., 1998).

The optical inaccessibility of the mammary fat pad has hampered efforts to image mammary epithelial cell behaviors at post-natal stages of development. Despite recent advances in deep tissue imaging (Dawson et al., 2021; Dawson and Visvader, 2021), quantitative analyses of dynamic cellular behaviors driving branching morphogenesis have not been reported in vivo or in explant culture. Much of our mechanistic understanding comes from analysis of branch elongation in stroma-free organoid cultures derived from pubertal or adult mammary glands. The tips of these branches are proliferative and multi-layered but lack the full myoepithelial coverage of TEBs in vivo (Ewald et al., 2008), although protocols where this coverage can be preserved have been reported (Nguyen-Ngoc et al., 2015; Caruso et al., 2022). Tracking epithelial cells in organoids has revealed that they engage in directional collective migration, but without breaching the basement membrane barrier (Ewald et al., 2012; Huebner et al., 2016). It has also been proposed that cell migration is coupled to radial cell intercalation to the surface epithelial layer to facilitate branch elongation (Neumann et al., 2018). Invasion of the tip is further propelled by mechanical constraint generated by myoepithelial cells behind the tip (Neumann et al., 2018).

The process of tip bifurcation is perhaps best understood based on live imaging studies on ex vivo cultured embryonic organs (Goodwin and Nelson, 2020; Lang et al., 2021). In the bifurcating kidney, differential morphogen receptor activation drives cell sorting movements between the branch point and daughter tips (Riccio et al., 2016). In developing lungs, proliferation is uniform in bifurcating tips, but oriented cell divisions and non-muscle myosin II (NMII)–mediated contractility of the actin cytoskeleton may contribute to bifurcations (Schnatwinkel and Niswander, 2013). Differentiation of mesenchyme-derived smooth muscle cells at the leading edge of the tip was suggested to create a cleft between daughter tips (Kim et al., 2015). However, genetic inhibition of smooth muscle differentiation does not abrogate lung branching morphogenesis (Young et al., 2020). In the stratified tip of the salivary gland, localized deposition of fibronectin into a wedge-like structure opens a narrow cleft between cells to split the tip (Sakai et al., 2003; Larsen et al., 2006). More recent studies suggest that branching is driven by expansion and mechanical buckling of the surface cell sheet resulting from the combination of strong cell–matrix adhesion and weak basal cell–cell adhesions (Wang et al., 2021).

Here, we employ long-term high-resolution live imaging to investigate the cellular mechanisms driving expansion of the mammary epithelial network through terminal branch elongation and tip bifurcation. To discern cell behaviors during these processes, we took advantage of an ex vivo culture system that preserves the native tissue architecture with its epithelial–mesenchymal interactions (Kratochwil, 1969; Voutilainen et al., 2012). Branching morphogenesis in this setup is consistent with morphometric analysis of embryonic mammary glands and includes both terminal bifurcation and side-branching events (Lindström et al., 2022 *Preprint*). For a comprehensive view and mechanistic understanding of cell behaviors contributing to epithelial branching and elongation, we utilized a non-lineage-specific approach to indiscriminately label and image the majority of embryonic mammary epithelial cells. Our findings suggest that branching morphogenesis is characterized by spatially coordinated parallel changes in cell cycle activity and cell motility within the tip, to alternate between terminal branch elongation and branch point generation. We also find that these behaviors coincide with extracellular signal-regulated kinase (ERK) signaling and NMII activity at the leading front.

## Results

### Proliferation is localized to the inner compartment and enriched in terminal tips

**Figure 1.**
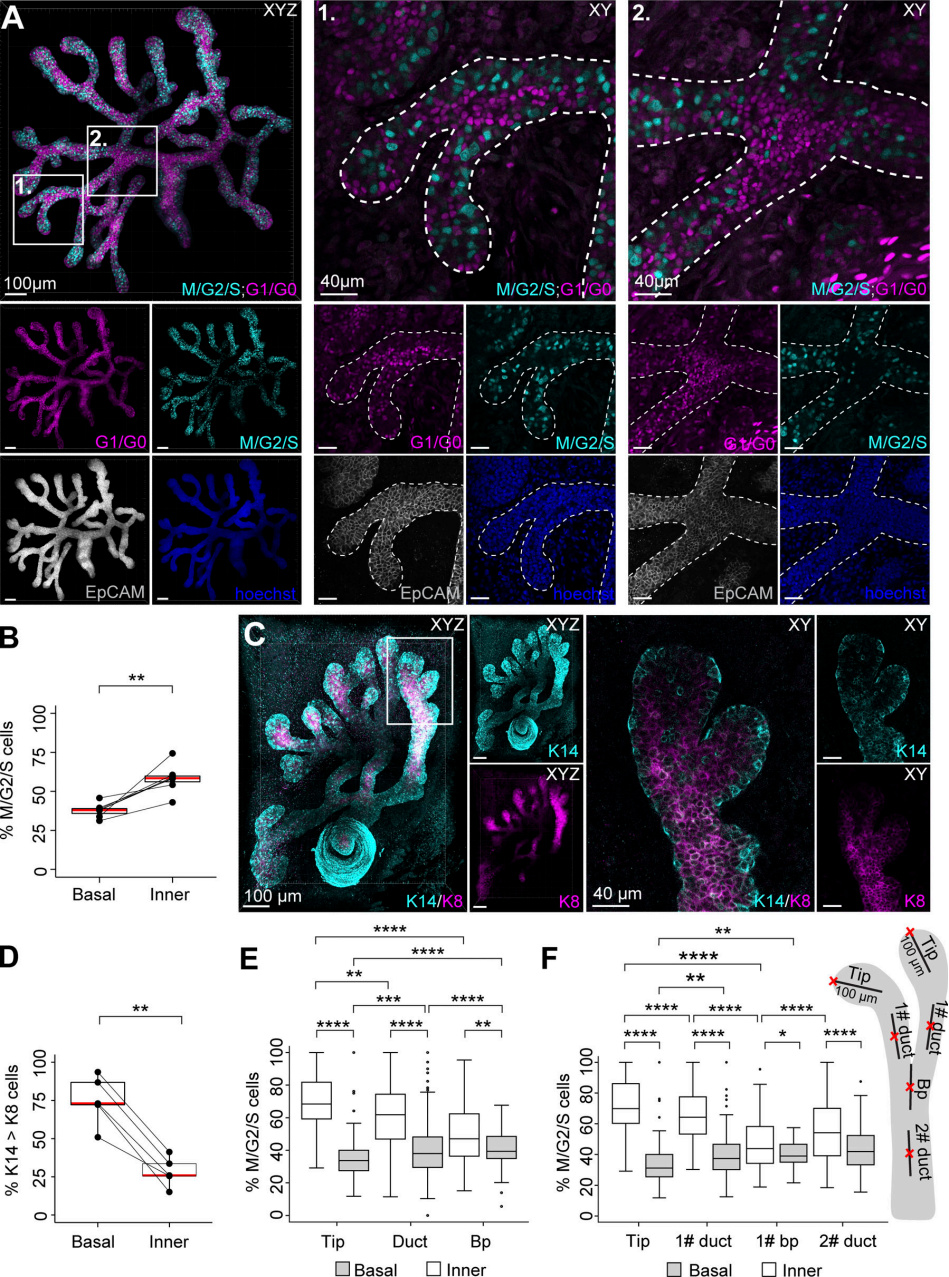
**Proliferation is localized to the inner compartment and enriched in terminal tips. (A)** Maximum intensity projection of the E18.5 mammary epithelium (left) with nuclear Fucci2a expression (M/G2/S; cyan, G1/G0; magenta) and EpCAM-staining (gray) used for epithelial surface rendering and masking of the mesenchyme. Cross-sections of two regions of interest are magnified on the right with both epithelial and mesenchymal signal visible: two terminal branches with their connecting branch point (1) and a proximal branch point (2). **(B)** Percentage of cells expressing the green M/G2/S cell phase reporter of all Fucci2a-labeled cells in the basal outermost cell layer (≤6 µm from the surface of the gland) and inner epithelial compartment (>6 µm from the surface of the gland; *n* = 7 glands of four embryos). **(C)** Maximum intensity projection of E18.5 mammary gland (left) with keratin 14 (K14, cyan) and keratin 8 (K8, magenta) immunofluorescence staining and a magnified cross-section of a terminal branch (right). **(D)** Quantification of the percentage of cells with more K14 than K8 expression in the basal and inner compartments (*n* = 5 glands of three embryos). **(E)** Percentage of cells in the M/G2/S phase in inner and basal compartments of terminal tips (at ≤100 µm distance from the terminal edge), ducts (within ≥50 µm radius of the estimated center point) and branch points (within ≥50 µm radius of the estimated center point; *n*^Tips^ = 121, *n*^Ducts^ = 216, *n*^Branch points^ = 94, from seven glands of four embryos). **(F)** Percentage of cells in the M/G2/S phase in the basal and inner compartments of terminal branches (≥150 µm in length, *n* = 74), with tips, ducts, branch points, and the segment of duct behind the branch point separately analyzed. Data shown represent the median (line) with 25th and 75th percentiles (hinges) plus 1.5× interquartile ranges (whiskers) with individual paired data-points in B and D illustrated by connecting lines. Statistical significance was assessed with the two-tailed paired *t* test (B and D), Wilcoxon’s rank sum test (E), and Wilcoxon’s signed rank test (F); *, P ≤ 0.05; **, P ≤ 0.01; ***, P ≤ 0.001; ****, P ≤ 0.0001.

**Figure S1.**
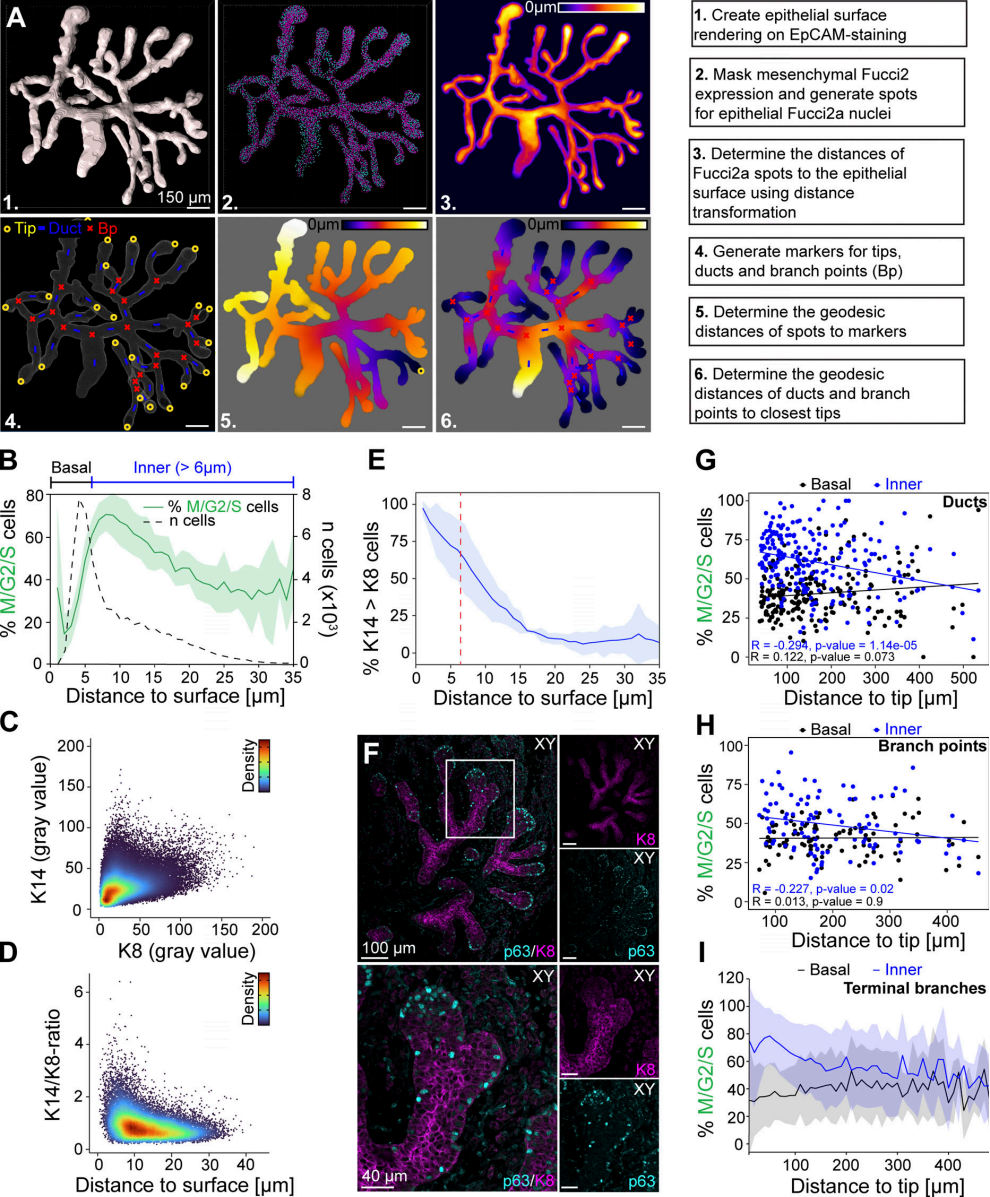
**Distribution of cell cycle indicators and cell fate markers in embryonic mammary glands. (A)** Pipeline for spatial analysis of cell cycle activity based on the Fucci2a reporter. **(B)** Distribution of cell cycle status (% M/G2/S cells) and cell density (*n* cells) by the distance to the surface of the gland from seven glands of four embryos. **(C and D)** Gray values of keratin 14 (K14) and keratin 8 (K8) per cell (C) and the ratio of K14-to-K8 gray values by the distance of the cell to the gland surface (D) derived from confocal 3D images of five E18.5 mammary glands of three embryos (*n* = 77,921 cells, color-coding indicates the density of cell neighbors; turbo LUT, blue→red = low→high). **(E)** The average % of cells with more K14 than K8 expression per gland (*n* = 5 glands of three embryos) by the distance from the gland surface. **(F)** Image of a paraffin section of a E18.5 mammary gland (top) and a close-up of a terminal branch (bottom), stained for p63 (cyan) and K8 (magenta). **(G)** Distribution of cell cycle status (% M/G2/S cells) in the basal (≤6 µm) and inner (>6 µm) compartment of ducts (sampled within ≥50 µm radius of the estimated center point) by the distance to the closest tip (*n* = 216 ducts from seven glands of four embryos). **(H)** Distribution of cell cycle status (% M/G2/S cells) in the “basal” and inner compartment of branch points (sampled within ≥50 µm radius of the estimated center point) by the distance to the closest tip (*n* = 94 branch points from seven glands of four embryos). **(I)** Distribution of basal and inner cell cycle status (% M/G2/S cells) along terminal branches, from the terminal edge to the branch point (*n* = 74 terminal branches ≥150 µm in length of seven glands, four embryos). Correlation between variables in G and H was assessed with the Pearson coefficient (R) and P value for the linear correlation given.

To investigate if localized cell proliferation contributes to embryonic mammary gland branching morphogenesis, we examined cell cycle status in fixed whole-mount glands at E18.5. We utilized a constitutive bicistronic fluorescent cell cycle reporter Fucci2a (Mort et al., 2014) and quantified cells in different phases of the cell cycle from 3D confocal images (Fig. 1 A). Cells preparing for mitosis (S/G2/M phase) express nuclear green fluorescent hGeminin-mVenus, while cells that exit mitosis (G1/G0 phase) express nuclear red fluorescent hCdt1-mCherry. Cells marked with either fluorophore constitute 75 ± 8 percent of all cells, as determined by manual counting of the cells from terminal segments of five different glands. For spatial analysis of whole glands, quantification was automated, and the cell cycle status was expressed as the percentage of S/G2/M cells at different locations (Fig. S1 A). We first examined cell cycle activity based on cell distance to the surface of the epithelium. The number of cells peaked at a 4–5 µm distance from the surface, as measured from the center of their nuclei, representing the position of the outermost cell layer (Fig. S1 B). Therefore, we considered cells with at ≤6 µm and >6 µm from the surface as basal and inner (“luminal”) cells, respectively. Surprisingly, we found that basal cells had a much higher G1/G0 cell cycle status overall compared to the inner cells (Fig. 1 B and Fig. S1 B). Quantification of the basal marker keratin 14 (K14) and luminal marker keratin 8 (K8) from whole-mount immunostainings revealed a bimodal distribution where cells often expressed one marker over the other (Fig. 1 C and Fig. S1 C). The ratio of K14-to-K8 per cell was higher the closer the cell was to the basal surface, indicative of a basal bias in K14 expression, as expected (Fig. S1 D). Indeed, the percentage of cells with more K14 than K8 expression was higher closer to the basal surface (Fig. S1 E), averaging 75% of cells in the basal compartment (Fig. 1 D). Also p63, a known basal cell fate specifying gene (Wuidart et al., 2016), was detected mainly in basal cells, although in the growing tips, also many inner cells were p63-positive (Fig. S1 F). Thus, although the expression patterns are biased, the cell fate markers are not yet mutually exclusive between compartments at E18.5.

Next, we assessed whether the cell cycle status was associated with any morphological domains of the mammary gland, and analyzed tips, ducts, and branch points separately (Fig. 1 A; see close-ups 1–2, Fig. S1 A). Inner cells displayed the highest cell cycle activity in the tips, followed by ducts, while branch points were the least active (Fig. 1 E). Conversely, cell cycle activity of basal cells was lowest at the tip and lower than inner cells in all the domains. As the network of ducts close to the tip are newly made, we explored whether cell cycle status changes with the distance to the closest tip. In both ducts and branch points, the proportion of S/G2/M inner cells gradually decreased the further away they were from the tips while the basal cells displayed high G1/G0 status regardless of their location (Fig. S1, G and H). To investigate if differences in cell cycle status were established already during branch elongation and branching events, we focused our analysis on terminal branches only (Fig. 1 A, see close-up 1). The proportion of S/G2/M inner cells peaked within a 100 µm distance from the leading edge of the tip, while basal cells were lower in mitotic activity throughout the length of the branch (Fig. S1 I). Tips had a significantly higher percentage of S/G2/M inner cells compared to the trailing duct and the latest branch point (Fig. 1 F and Fig. S1 I). Notably, branch points were even less mitotic than the segment of duct immediately behind the branch point. Taken together, cell cycle analysis of late embryonic mammary glands revealed that inner cells are distally proliferative, while basal cells are relatively less proliferative throughout the gland. Our data also suggest that inner cells in the tips can be discerned from the latest branch point—a potential bifurcation site—by a much higher proliferation rate.

### Tips proliferate during branch elongation, while branch points become cell cycle repressed upon tip bifurcation

**Figure 2.**
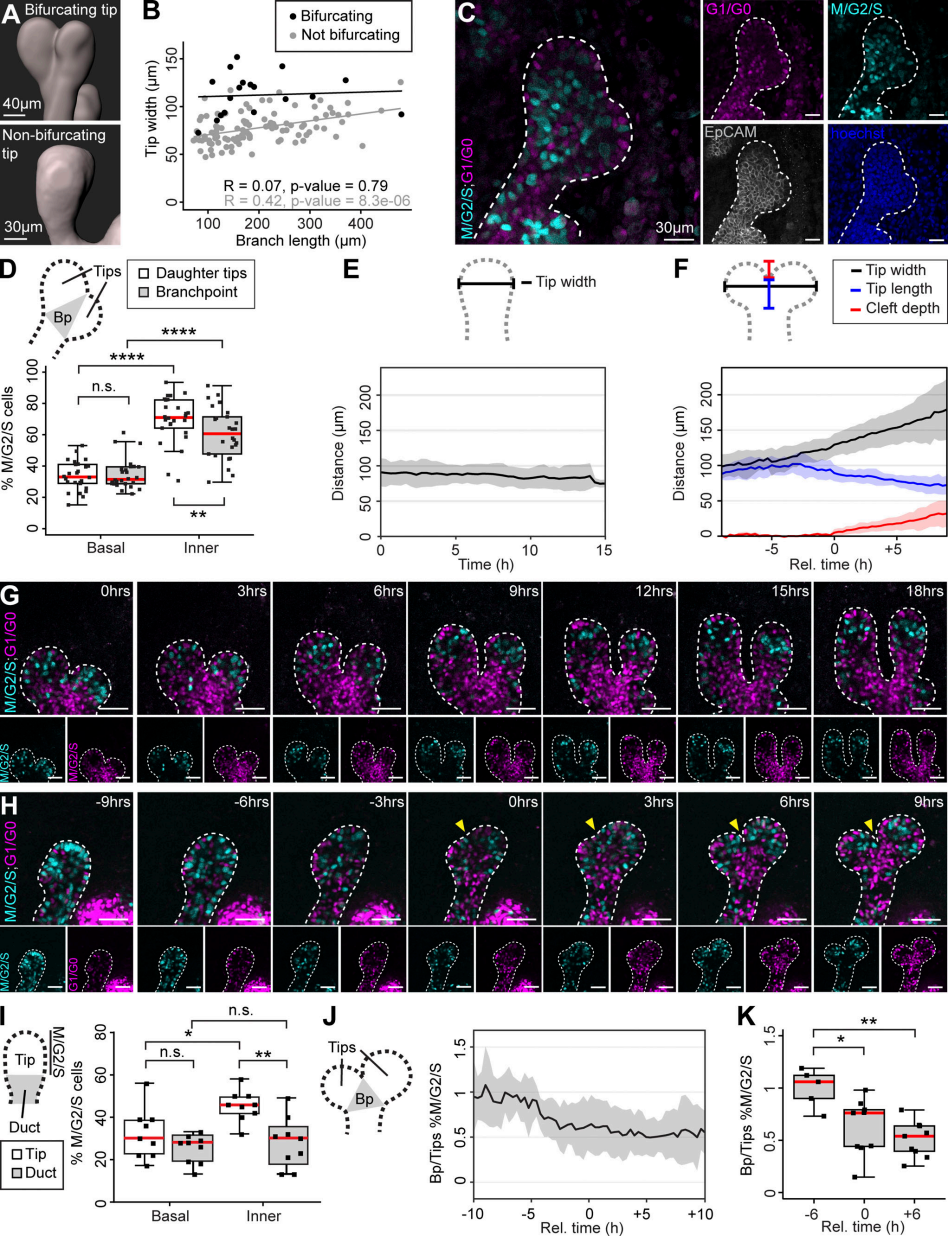
**Tips proliferate during branch elongation, while branch points become cell cycle repressed upon tip bifurcation. (A)** Epithelial surface rendering of a bifurcating tip (top) and a non-bifurcating tip (below) generated based on whole-mount EpCAM staining. **(B)** Analysis of tip width by branch length in terminal branches of the surface rendered E18.5 mammary gland with bifurcating and non-bifurcating tips (*n*^bifurcating^ = 18, *n*^non-bifurcating^ = 103 tips of seven glands, four embryos). **(C)** Confocal slice across a bifurcating tip and surrounding mesenchyme with Fucci2a expression (M/G2/S; cyan, G1/G0; magenta) and EpCAM-staining (gray). **(D)** Quantification of cell cycle status in the basal and inner compartment of bifurcating tips based Fucci2a fluorescent nuclei, comparing the emerging daughter tips to the branch point as delineated in the schematic (*n* = 26 bifurcating tips, four embryos; the branch point was defined as a triangular region running from the cleft to the necks of the daughter tips, as shown in the schematic). **(E)** Confocal time-lapse imaging was performed on ex vivo cultured mammary glands with epithelial Fucci2a expression (*K14-Cre;R26R-Fucci2a-flox/wt*), and tip width was measured as shown in the schematic on top from elongating terminal branches, based on an epithelial surface rendering (*n* = 9 branches, four experiments). **(F)** Dimensions of the bifurcating tip were measured as indicated in the schematic from time-lapse videos at different time points before and after the appearance of a cleft (T0; *n* = 10 bifurcations, seven experiments). **(G)** Captions of a time-lapse imaging series featuring two elongating branches with epithelial Fucci2a expression (size bar = 50 µm). Note the enrichment of M/G2/S phase nuclei in the tips, which was used as a proxy to define the tip border in the absence of a discernable neck. **(H)** Captions featuring a bifurcating tip with epithelial Fucci2a expression (yellow arrowhead points to the cleft, size bar = 50 µm). **(I)** Quantification of cell cycle status (% M/G2/S phase nuclei) from confocal time-lapse imaging videos of elongating branches comparing the tip to the trailing duct with basal and inner compartments analyzed separately (*n* = 9 branches, four experiments, data was pooled from all time points). **(J)** The relative cell cycle status between the branch point (defined as the triangular region running from the cleft to the necks of the emerging daughter tips) and the daughter tips (the branch point-to-tip ratio of % M/G2/S nuclei) of time-lapse imaged bifurcations was plotted against time normalized according to the appearance of the cleft (T0; *n* = 9 bifurcating tips, six experiments). **(K)** The ratio of % M/G2/S nuclei was compared 6 h before, upon (T0), and 6 h after the appearance of the cleft in different videos (*n* = 9 bifurcating tips, six experiments). Correlation between variables in B was assessed with the Pearson coefficient (R) and P value for the linear correlation given. Data shown in D, I, and K represent the median (line) with 25th and 75th percentiles (hinges) plus 1.5× interquartile ranges (whiskers). Data shown in E, F, and J represent average (line) ± SD (shaded region). Statistical significance was assessed with the Wilcoxon’s signed rank test (D) and paired two-tailed *t* test (I and K); *, P ≤ 0.05; **, P ≤ 0.01; ***, P ≤ 0.001; ****, P ≤ 0.0001.

To examine bifurcating tips in detail, we identified tips where a cleft could be unequivocally detected in our dataset of fixed E18.5 mammary glands (Fig. 2 A). We first asked whether branches with bifurcating tips have a characteristic tip width or branch length, compared to branches with non-bifurcating tips, i.e., potentially elongating branches. Non-bifurcating tips were wider the longer the branch (Fig. 2 B), suggesting that tips widen as branches elongate in vivo. The width of the bifurcating tips was greater than non-bifurcating tips, irrespective of branch length, suggesting that tip widening is associated with tip bifurcation. Bifurcating tips were found in branches of all length, in line with the common notion that branching of the mammary gland is non-stereotyped (Goodwin and Nelson, 2020; Scheele et al., 2017; Lindström et al., 2022 *Preprint*). To investigate if tip bifurcation involves local changes in cell cycle status, we quantified the Fucci2a cell cycle reporters in bifurcating tips, comparing the nascent daughter tips to the branch point in between (Fig. 2 C). We found that inner cells in the branch point had a significantly reduced S/G2/M status compared to the daughter tips (Fig. 2 D), similar to branch points in terminal branches (Fig. 1 D). In contrast, the cell cycle status of the basal cells did not differ.

**Figure S2.**
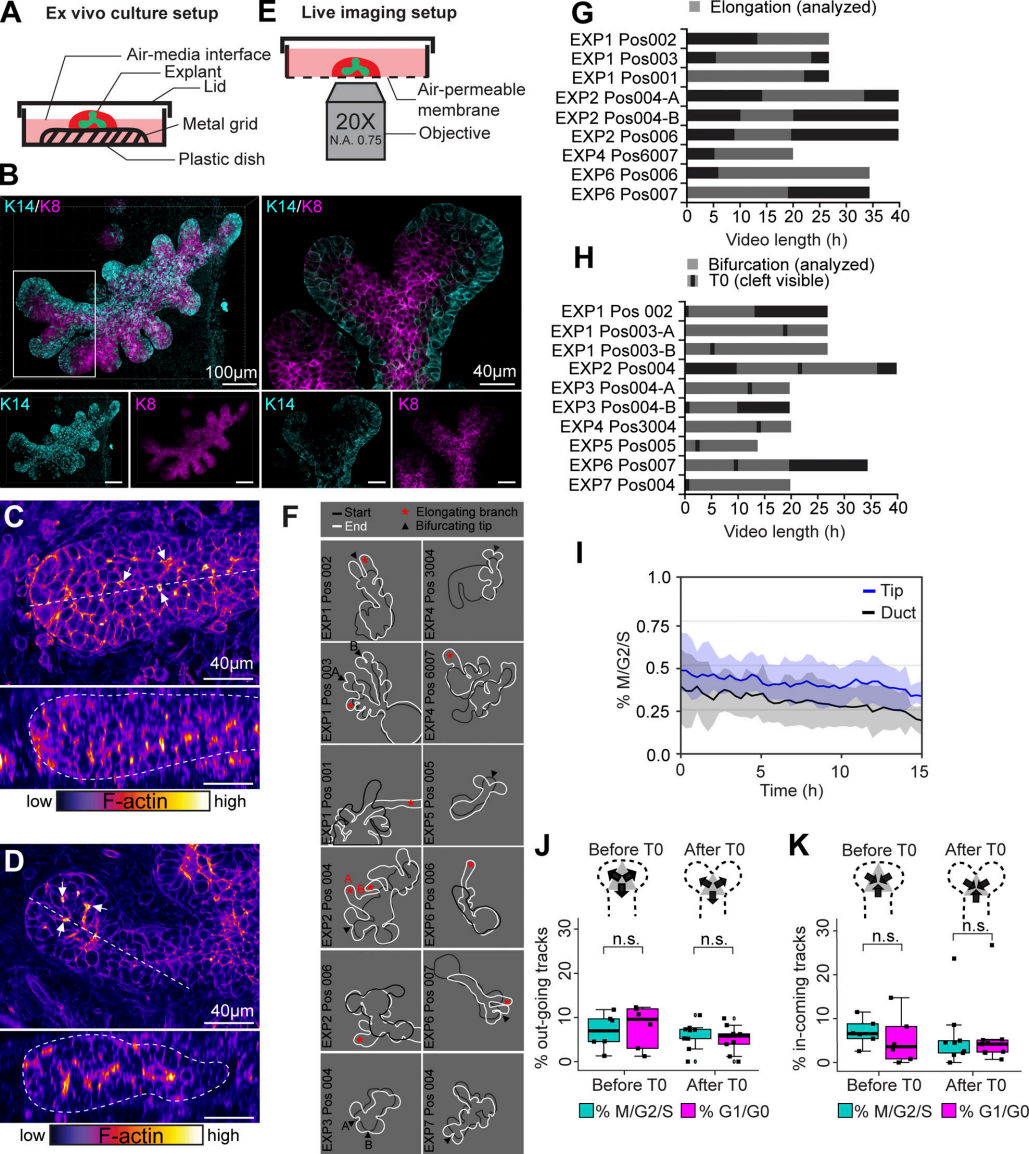
**Ex vivo culture system for live cell imaging of cell cycle indicators in embryonic mammary glands. (A)** Depiction of the ex vivo culture setup for the embryonic mammary gland. **(B)** Whole-mount immunofluorescence staining of a 5-d cultured mammary explant (left) and a confocal slice of a bifurcating terminal branch (right), stained for K14 (cyan) and K8 (magenta). **(C and D)** The presence of microluminae (white arrows) in embryonic mammary branches in vivo (C) and ex vivo (D) was assessed based on F-actin staining with phalloidin (fire LUT, black→white = low→high). **(E)** The picture indicates how explants, first cultured as shown in A, were mounted for confocal live imaging. **(F)** Outlines of the ex vivo cultured glands at the beginning (black line) and at the end (white line) of time-lapse imaging, where analyzed elongating terminal branches (marked by a red star) and tip bifurcations (black arrowhead) are indicated. Data was collected from seven imaging sessions and 12 different glands. **(G)** The length of videos that were captured, where the period of visible branch elongation was used for analysis (gray). **(H)** The length of videos with bifurcating tips (analyzed period in gray), where the timing of different bifurcations was normalized based on the appearance of a cleft (black line; T0). **(I)** The effect of long-term time-lapse imaging on the cell cycle activity (% M/G2/S cells) as a function of time, visualized in terminal branches (*n* = 9, four experiments). **(J)** The percentage of M/G2/S and G1/G0 cells that moved out of the branch point during tip bifurcation (*n* = 9, six experiments). **(K)** The percentage of M/G2/S and G1/G0 cells that moved into the branch point during tip bifurcation (*n* = 9, six experiments). Data shown in J and K represent the median (line) with 25th and 75th percentiles (hinges) plus 1.5× interquartile ranges (whiskers). Data shown in I represents average (line) ± SD (shaded region). Statistical significance was assessed with two-tailed paired *t* test.

To examine branch elongation and tip bifurcation in real time, we performed live imaging of ex vivo cultured mammary glands derived from embryos expressing the Fucci2a reporter only in epithelial cells (*K14-Cre;R26R-Fucci2a-flox/wt* mice). We utilized an established protocol, where E13.5 mammary buds isolated with their surrounding mesenchyme, initiate branching morphogenesis on day 3 of culture (Fig. S2 A; Voutilainen et al., 2012; Lan et al., 2022). On day 5 of culture, mammary rudiments had undergone several rounds of branching and displayed a characteristic basal bias in K14/K8 expression (Fig. S2 B), similar to E18.5 mammary glands in vivo (Fig. 1 C). The presence of microluminae that begin to emerge at ∼E17.5 (Hogg et al., 1983) was confirmed in the elongating tips of both 5-d cultured explants and E18.5 mammary glands (Fig. S2, C and D). Confocal whole-mount imaging was performed on day 5–6 (Fig. S2 E) and cells were imaged in elongating branches and in branches undergoing terminal bifurcation for up to 28 h at 20-min intervals (Fig. S2, F–H; and Videos 1 and 2). We first studied the morphometrics of the tip based on epithelial 3D surface rendering, created around all the fluorescent nuclei. Tips that did not bifurcate, maintained a constant width over time while elongating (Fig. 2 E). Tips that bifurcated increased in width before a cleft became visible (Fig. 2 F). At the same time, the neck of the tip became more pronounced, and the tip flattened as measured from the neck to the leading front.

**Video 1.** **Elongating branch followed by time-lapse confocal microscopy in an ex vivo cultured mammary gland expressing Fucci2a dual nuclear cell cycle reporter (M/G2/S = cyan, G1/G0 = magenta).** 3D images were collected every 20 min for a total of 15 h (related to Fig. 2 G, display rate = 3 frames/s). Time-lapse is shown as maximum intensity projection (MIP), a confocal slice through the middle of the branch, and finally with automated nuclear detection overlayed with the MIP.

**Video 2.** **Bifurcating branch followed by time-lapse confocal microscopy in an ex vivo cultured mammary gland expressing Fucci2a dual nuclear cell cycle reporter (M/G2/S = cyan, G1/G0 = magenta).** 3D images were collected every 20 min for a total of 18 h (related to Fig. 2 H, display rate = 3 frames/s). Time-lapse is shown as maximum intensity projection (MIP), a confocal slice through the middle of the branch, and finally with automated nuclear detection overlayed with the MIP.

To study cell cycle dynamics, Fucci2a fluorescent nuclei were automatically detected as spots and quantified. Consistent with our in vivo findings, tips of elongating branches appeared to be populated with S/G2/M phase inner cells (Fig. 2 G and Video 1), which we used as a proxy to distinguish the tip from the more G1/G0 positive trailing duct. We noticed that prolonged imaging had a negative effect on cell cycle activity over time, but the difference between the tip and the trailing duct persisted throughout the imaging (Fig. S2 I). The aggregated time-lapse data from all the videos confirmed the enrichment of proliferative inner cells in the tip, consistent with our in vivo findings (Fig. 2 I). To assess if the cell cycle status changes within the tip as tips bifurcate (Fig. 2 H and Video 2), we determined the proportion of S/G2/M cells in branch points and daughter tips and plotted their ratio as a function of time, where the time point of cleft appearance was defined as T0 (Fig. 2 J). The branch point-to-tip ratio decreased substantially prior to T0 and remained low thereafter (Fig. 2, J and K). Although these results point towards a localized cell cycle repression, cells may also move in and out of the branch point in a cell cycle dependent manner. To investigate this possibility, we compared the proportion of incoming and exiting S/G2/M and G1/G0 cells but found no difference (Fig. S2, J and K).

Taken together, our real time cell cycle analysis of ex vivo cultured embryonic mammary glands indicates that elongating branches maintain a pool of S/G2/M inner cells at the tip. As the tip bifurcates, cells at the branch point become cell cycle repressed, while the newly formed daughter tips remain proliferative.

### Inhibition of cell proliferation does not prevent branch elongation or branch point generation

**Figure 3.**
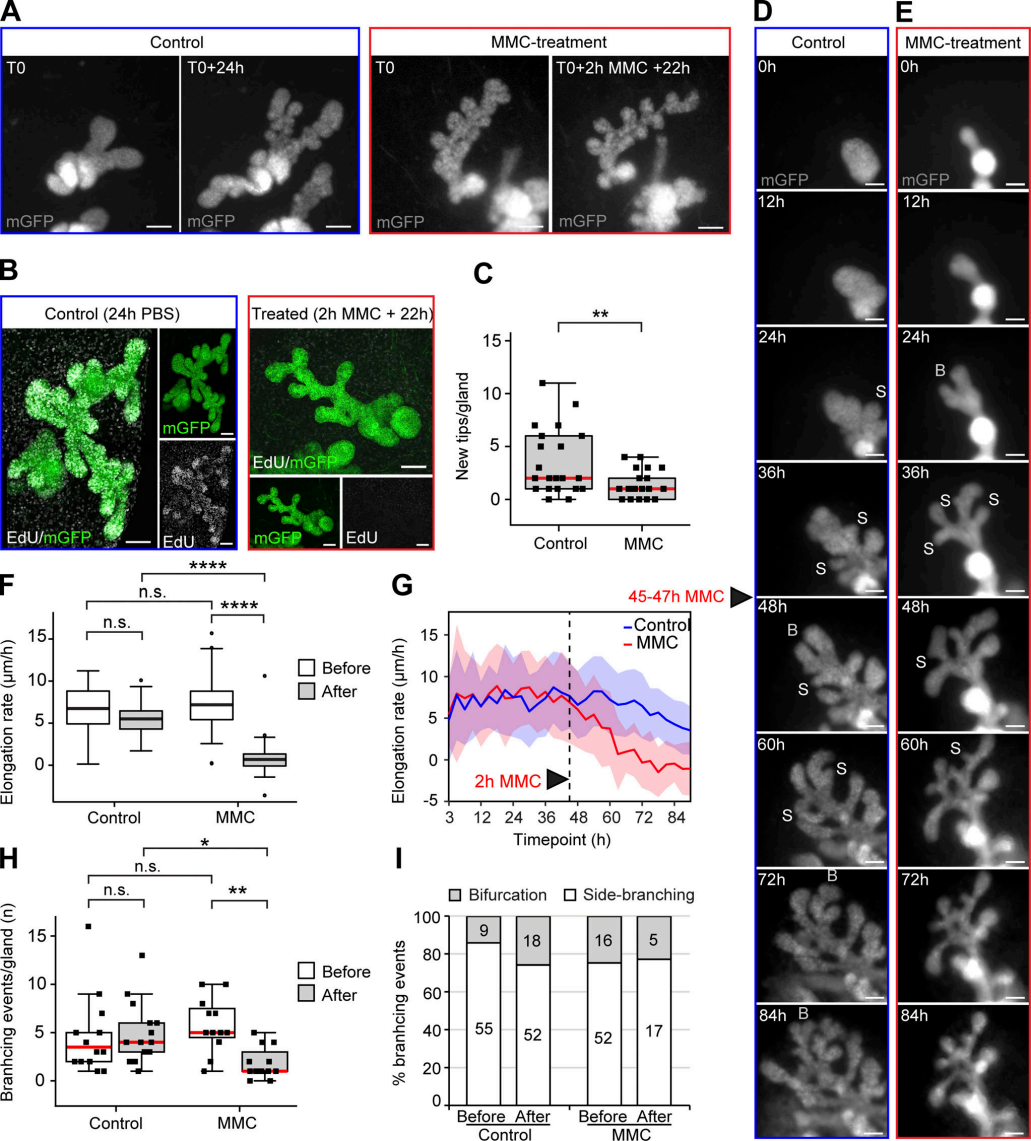
**Inhibition of cell proliferation does not prevent branch elongation or branch point generation. (A)** Ex vivo cultured mammary glands with epithelial mGFP expression (*K14-Cre*;*R26R-mTmG*), were imaged before and after 2 h treatment with 1 µg/ml of Mitomycin C (MMC) for cell cycle repression or vehicle (control) followed by 22 h of culture in normal media (mGFP = gray). **(B)** To assess the effects of MMC treatment on cell proliferation, control and treated glands were labeled with EdU for 1 h at the end of the culture period and fixed for staining and confocal imaging (maximum intensity projections of EdU in white and mGFP in green). **(C)** The number of tips was quantified from wide-field images of the mGFP-labeled epithelium (A) before and after culturing (*n*^control^ = 21 glands from 10 explants, *n*^treated^ = 22 glands from 12 explants; four experiments). The effects of cell cycle repression on branching morphogenesis was studied by wide-field time-lapse imaging of epithelial mGFP expressing explants (93 h of imaging with 3-h intervals). **(D and E)** In the middle of the time-lapse (45–47 h), explants were treated for 2 h either with vehicle (D) or with MMC (E), followed by imaging in normal media. **(F)** The length of terminal branches was measured as a function of time and the elongation rate (Δ length/time) between control and treated glands, before and after treatment compared (*n*^control^ = 73 branches from 13 glands, *n*^treated^ = 62 branches from 12 glands; two time-lapse experiments). **(G)** The rate of elongation between adjacent time points was determined for each branch and the average plotted as a function of time. **(H)** Branching events were scored in each gland by careful observation of time-lapse videos and the number of events that occurred in control and treated glands before and after treatment compared (*n*^control^ = 13 glands, *n*^treated^ = 12 glands). The branching events could be further identified as bifurcations or side-branching events as shown in D and E. **(I)** To assess their prevalence, the overall percentages of different types of branching events were calculated by pooling of events from different glands (total number of events indicated in the chart). Data shown in C, F, and H represent the median (line) with 25th and 75th percentiles (hinges) plus 1.5× interquartile ranges (whiskers). Data shown in G represents average (line) ± SD (shaded region). Statistical significance was assessed with the Wilcoxon’s rank sum test (C), two-tailed paired *t* test (F), or Wilcoxon’s rank sum test (H); *, P ≤ 0.05; **, P ≤ 0.01; ****, P ≤ 0.0001. Size bars = 100 µm.

Cell proliferation is required for growth of the mammary epithelium, but whether cell divisions per se can elongate or initiate branches during branching morphogenesis, remains unclear. To address this question, we examined the effects of global cell cycle inhibition on branching morphogenesis by utilizing a short 2-h ex vivo treatment of Mitomycin C (MMC) followed by culture in fresh medium for 24 h using explants expressing epithelial GFP (*K14-Cre*;*R26R-mT/mG*) for easy visualization (Fig. 3 A). EdU labeling at the end of the culture period confirmed that cell proliferation had been suppressed (Fig. 3 B). Next, we quantified the number of new branches generated over the 24-h culture after MMC treatment. As expected, the number of new branches was much lower than in non-treated control explants (Fig. 3 C).

**Figure S3.**
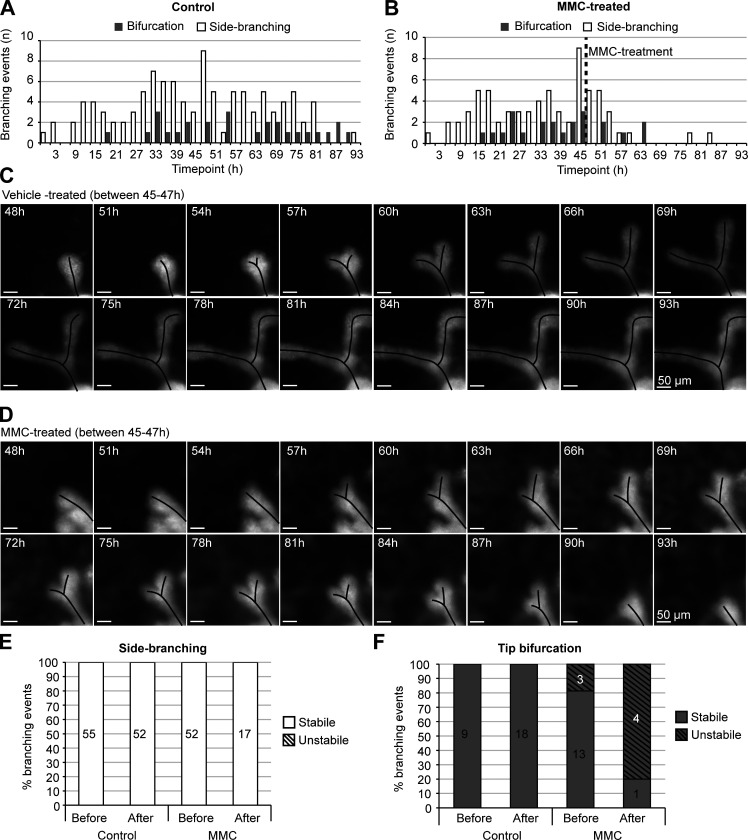
**Bifurcations are unstable under cell cycle repression. (A)** Occurrence of tip bifurcation and side-branching events in each time point of the time-lapse imaging experiment, where glands were treated with vehicle between 45 and 47 h (*n*^before^ = 64, *n*^after^ = 70 from 13 glands, two experiments). **(B)** Occurrence of tip bifurcation and side-branching events in each time point of the time-lapse imaging experiment, where glands were treated with 1 µg/ml of MMC between 45 and 47 h (*n*^before^ = 68, *n*^after^ = 22 from 12 glands, two experiments). **(C)** Captions from a time-lapse imaging video, focusing on a tip bifurcation from a control gland cultured in normal conditions. **(D)** Captions from a time-lapse imaging video, focusing on an unstable tip bifurcation that initiated after MMC treatment. **(E)** Side-branching events were evaluated based on whether or not they were maintained once initiated (*n* indicated in figure). **(F)** Tip bifurcations were evaluated based on whether or not they were maintained once initiated (*n* indicated in figure).

To analyze where and when the effects of MMC were manifested, we performed low-resolution time-lapse imaging of cultured mammary explants for a total of 93 h, taking pictures every 3 h (Fig. 3, D and E; and Videos 3 and 4). A 2-h MMC treatment was administered to half of the explants at 45 h, when several branches had already formed. We first analyzed elongation of branches by measuring branch length over time. The rate of elongation was significantly reduced in MMC-treated samples compared to control samples (Fig. 3 F). More detailed analysis revealed that branch elongation did not cease completely until 16 h after the treatment (Fig. 3 G). This finding prompted us to analyze the role of cell divisions in initiation of branch formation. As tip bifurcation and side-branching are essentially different forms of tissue morphogenesis (Wang et al., 2017), we wanted to investigate how cell divisions contribute into each. As expected, time-lapse imaging revealed that MMC treatment reduced branching events (Fig. 3 H), yet the ratio of tip bifurcation and side-branching events remained unchanged (Fig. 3 I). Most of the remaining branching events occurred within 16 h of the MMC treatment (Fig. S3, A and B), suggesting that branching beyond this point is limited by exhaustion of new cells. Interestingly, we noticed a difference in the stability of side branches and bifurcations. Side branches were stable throughout imaging, while tip bifurcations often returned to their unbifurcated state (Fig. S3, C–F).

**Video 3.** **Branching morphogenesis in an ex vivo cultured mammary gland expressing epithelial mGFP imaged by time-lapse epifluorescence microscopy.** 2D images were collected every 3 h for a total of 93 h and between 45 and 47 h, explants were treated with media containing a vehicle control (PBS), followed by imaging in normal media. Frame display rate = 2 frames/s.

**Video 4.** **Branching morphogenesis in an ex vivo cultured mammary gland expressing epithelial mGFP imaged by time-lapse epifluorescence microscopy.** 2D images were collected every 3 h for a total of 93 h and between 45 and 48 h, explants were treated with media containing 1 µg/ml of MMC, followed by imaging in normal media. Frame display rate = 2 frames/s.

Based on imaging the effects of cell cycle repression in ex vivo culture, we found that branching morphogenesis is progressively limited by cell number. In particular, cell proliferation is required to sustain two daughter tips after a tip bifurcation has taken place.

### Epithelial cell contractility is essential for mammary gland branching morphogenesis

**Figure 4.**
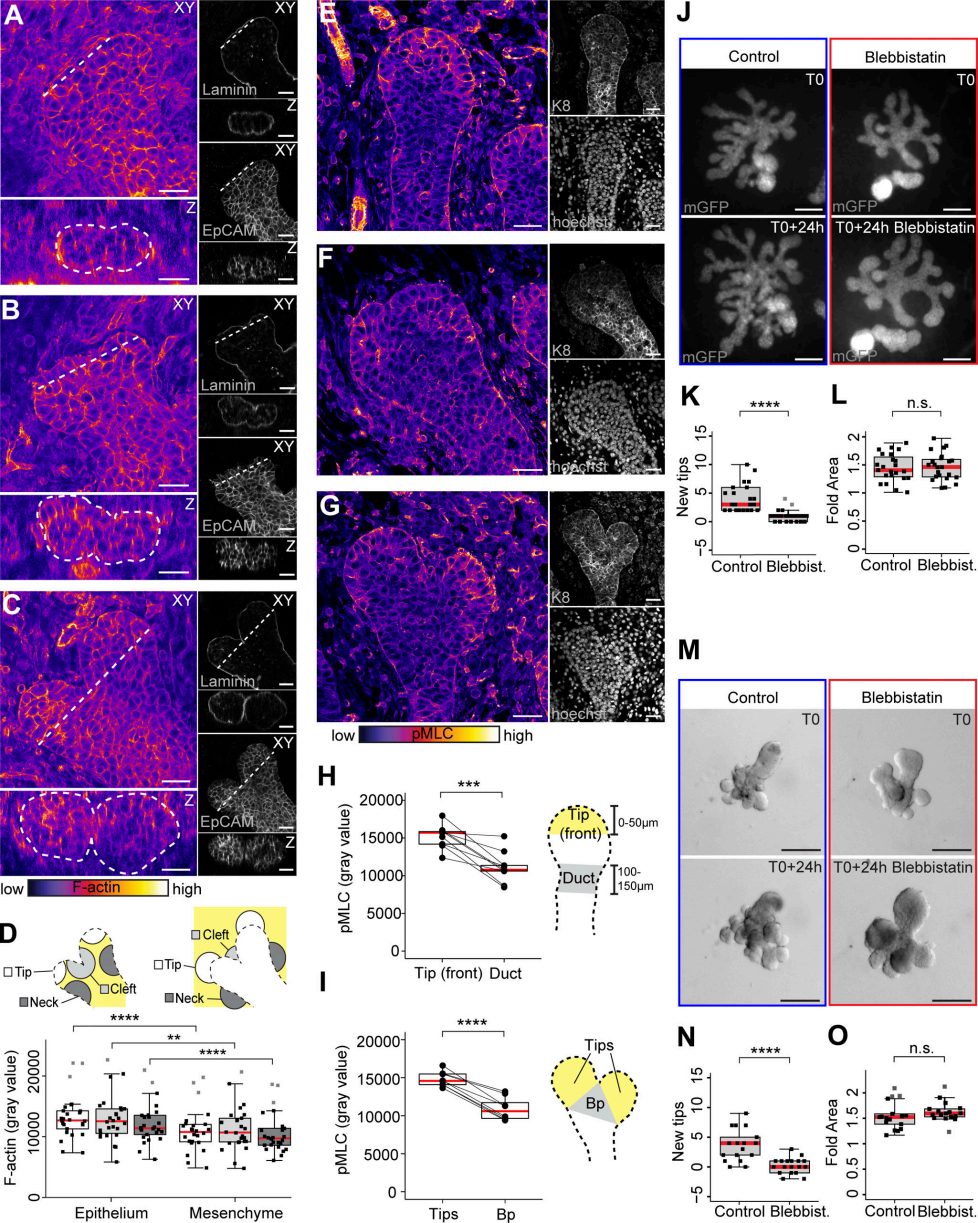
**Epithelial cell contractility is essential for mammary gland branching morphogenesis. (A)** Confocal xy-slice of a tip at an early stage of bifurcation, stained with F-actin- (fire LUT, black→white = low→high), laminin (gray) and EpCAM (gray). A vertical cross-section of the tip along the dotted line in the xy-view is shown below. **(B and C)** Similar images of an early bifurcating tip with a more obvious cleft (B) and late bifurcating tip, where cleft has progressed further (C). **(D)** Quantification of F-actin intensity within epithelial and mesenchymal regions around the daughter tip, cleft and neck of the bifurcating tip as marked in the schematic (*n* = 26, from 16 glands of seven embryos). **(E)** Image of a non-bifurcating tip from paraffin sections, stained with anti-pMLC (fire LUT, black→white = low→high) and anti-K8 (gray) antibodies together with Hoechst (gray). **(F and G)** Bifurcating tips with early (F) and more advanced clefts (G) were also imaged from similarly stained sections. **(H and I)** The intensity of pMLC staining (gray value) was quantified from non-bifurcating (H) and bifurcating branches (I) as shown in the corresponding schematics on the left (*n*^non-bifurcating^ = 10 branches from two embryos, *n*^bifurcating^ = 9 branches from two embryos). **(J)** Ex vivo cultured mammary glands with epithelial mGFP expression (*K14-Cre*; *R26R-mTmG*), were imaged before and treatment with 20 µg/ml of blebbistatin or vehicle (mGFP = gray). **(K and L)** The number of new tips was quantified (K) and the growth area measured (L) from wide-field images of the mGFP-labeled epithelium before and after treatment (*n*^control^ = 22 glands from 10 explants, *n*^treated^ = 22 glands from 10 explants; two experiments) and increase in growth area presented as fold change. **(M)** Mammary epithelial rudiments cultured in 3D Matrigel were imaged before and after treatment with 20 µM blebbistatin or vehicle (brightfield). **(N and O)** The number of tips was quantified (N) and the growth area measured (O) from brightfield images before and after treatment (*n*^control^ = 17 glands, *n*^treated^ = 18 glands, from two experiments), and growth area presented as fold change. Data shown represents the median (line) with 25th and 75th percentiles (hinges) plus 1.5× interquartile ranges (whiskers). Statistical significance was assessed with the Wilcoxon’s signed rank test (D), two-tailed paired *t* test (H and I), Wilcoxon’s rank sum test (K and L) or two-tailed Welch’s *t* test (N and O); **, P ≤ 0.01; ****, P ≤ 0.0001. Size bars in A–C and E–G = 30 µm, in J = 200 µm, and in M = 100 µm.

**Figure S4.**
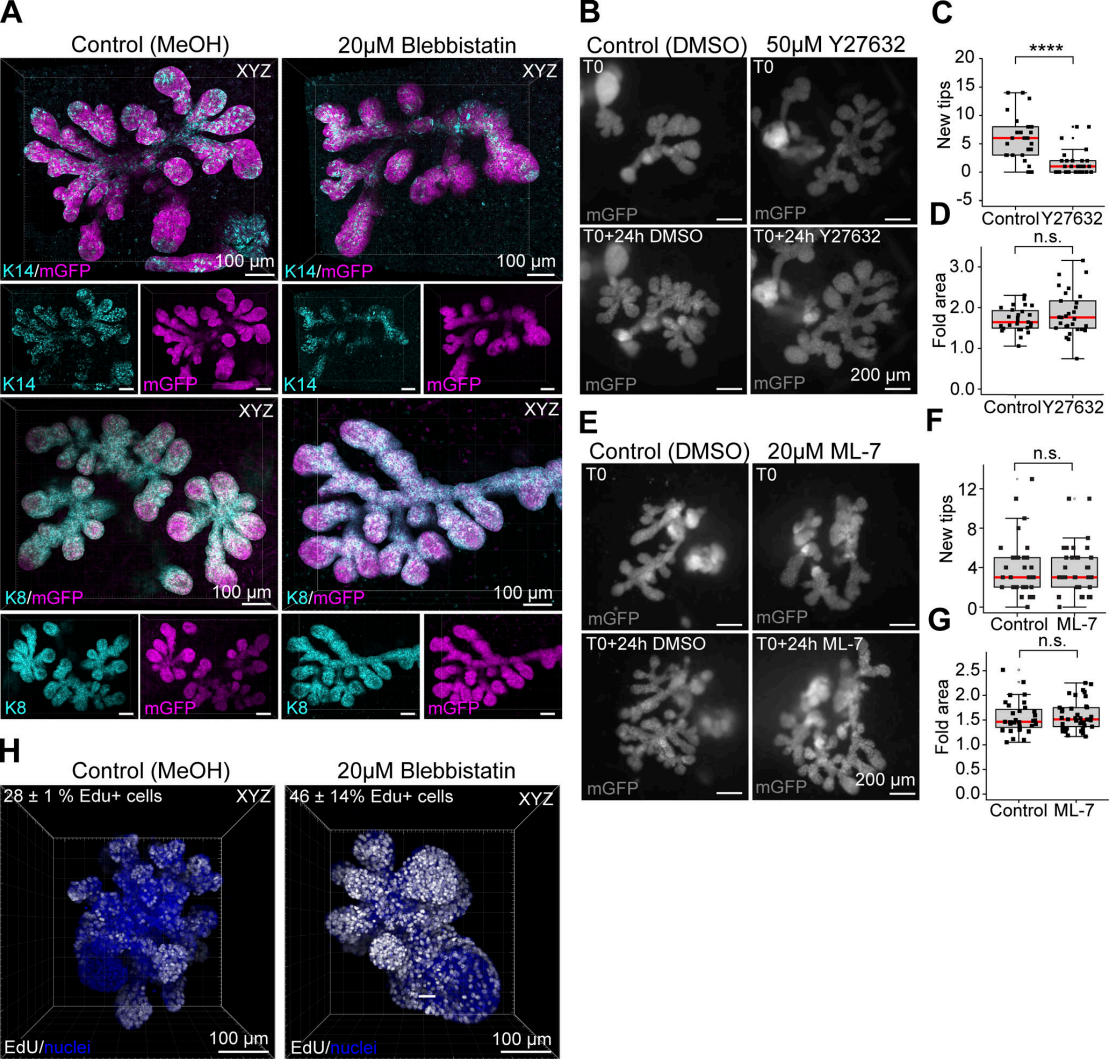
**Ex vivo inhibitor treatments implicate RhoA/ROCK pathway in NMII activation during branching. (A)** Explants derived from *K14-Cre*;*R26R-mTmG* embryos were cultured for 5 d and treated 24 h with 20 µM blebbistatin (right) or vehicle (left) and after whole-mount staining of K14 (cyan, upper pair of glands) or K8 (cyan, lower pair of glands) visualized together with epithelial mGFP (magenta) by confocal imaging. **(B and E)** To assess the roles of ROCKI/II and MLCK, ex vivo cultured mammary glands with epithelial mGFP expression (*K14-Cre*; *R26R-mTmG*), were also imaged before and after treatment with 50 µM Y27632 (B) or 20 µM ML-7 (E) together with vehicle (mGFP = gray). **(C, D, F, and G)** The number of new tips was quantified (C and F), and the fold increase in growth area measured (D and G) from wide-field images of the mGFP-labeled epithelium before and after treatment (*n*^control for Y27632^ = 27 glands from 13 explants, *n*^Y27632^ = 29 glands from 13 explants, four experiments; *n*^control for ML-7^ = 31 glands from 15 explants, *n*^ML-7^ = 37 glands from 15 explants, four experiments). **(H)** To assess the effects of blebbistatin treatment on cell proliferation, control and treated mesenchyme-free sprouting organoids were labeled with EdU for 1 h at the end of the 24-h culture period, fixed for staining and confocal imaging (maximum intensity projections of EdU in white and Hoechst in blue). The percentage of EdU+ cells is indicated on the top left corner (*n*^control^ = 3 rudiments, *n*^treated^ = 2 rudiments, one experiment. Data shown represents the median (line) with 25th and 75th percentiles (hinges) plus 1.5× interquartile ranges (whiskers). Statistical significance was assessed with the Wilcoxon’s rank sum test; ****, P ≤ 0.0001.

An important regulator of epithelial shaping is the contractile actomyosin network (Miao and Blankenship, 2020). To investigate if tip bifurcations utilize contractility, we examined filamentous actin within regions of high epithelial curvature (Fig. 4, A–C). Based on our measurements, there were no significant differences in intensity between three gross areas: around the cleft, daughter tips, and neck (Fig. 4 D), although staining was slightly elevated in the epithelium compared to the mesenchyme. We also observed no localized F-actin mediated tissue constrictions in tips, varying from early to later stages of bifurcation (Fig. 4, A–C). As not all actin filaments may be contractile, we investigated the localization of phosphorylated myosin light chain (pMLC). Strikingly, pMLC was found to be significantly enriched towards the leading front of non-bifurcating tips and newly bifurcated daughter tips in contrast to the branch point region (Fig. 4, E–I). This suggests that actomyosin contractility may be involved in facilitating cell behaviors at the leading front, rather than at the cleft region as we had initially predicted. To functionally assess the role of actomyosin contractility, we inhibited the ATPase activity of NMII in ex vivo culture by treating mammary explants with blebbistatin over a 24-h period (Fig. 4 J). The results show that branching morphogenesis, determined by the number of new tips, was strongly inhibited by blebbistatin, yet growth of the epithelium, assessed by the fold increase in epithelial area, was not affected (Fig. 4, K and L). Distribution of the basal marker K14 and luminal marker K8 were similar in control and treated glands, suggesting that cell fates are not overtly affected (Fig. S4 A). To study how NMII is activated under these conditions, we inhibited the phosphorylation of its regulatory light chain by Rho-associated protein kinases (ROCK) I and II or by MLC kinase (MLCK), using Y-27632 and ML-7, respectively. Under ROCK-inhibition, fewer tips formed in mammary explants (Fig. S4, B and C) without a significant impact on growth (Fig. S4 D). No such role was confirmed for MLCK with the ML-7 concentrations we tested (Fig. S4, E–G). To distinguish whether contractility is required in the epithelium or mesenchyme, we inhibited NMII activity with blebbistatin in mesenchyme-free sprouting organoids using a novel technique where intact E16.5 mammary epithelial rudiments are cultured in Matrigel in stroma-free conditions (Lan et al., 2022). Sprouting organoids were cultured for 2 d, followed by a 24-h treatment with blebbistatin (Fig. 4 M). The results show that blebbistatin strongly inhibited branching of embryonic mammary epithelial rudiments (Fig. 4 N). Again, epithelial growth was unobstructed as determined based on quantification of fold increase in epithelial area and EdU incorporation (Fig. 4 O and Fig. S4 H). These results indicate that mammary gland branching morphogenesis is dependent on epithelial actomyosin contractility, mediated by the RhoA (ras homolog family member A) /ROCK pathway, most likely regulating cell behaviors at the leading fronts of tips.

### Differential cell motility creates a retrograde flow of cells during branch elongation, which is restricted upon tip bifurcation

**Figure 5.**
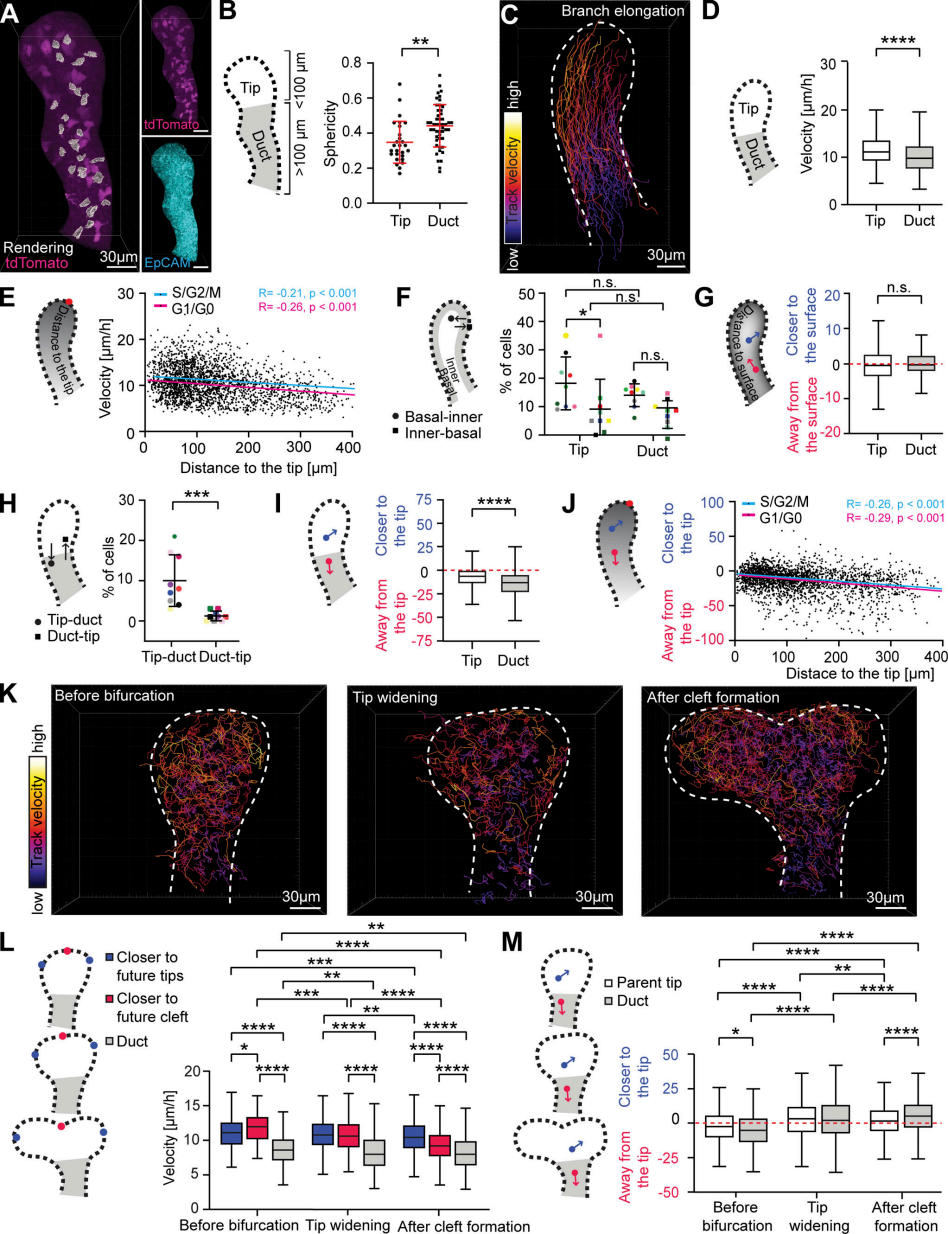
**Differential cell motility creates a retrograde flow of cells during branch elongation, which is restricted upon tip bifurcation. (A)** Maximum intensity projection of sparsely labeled tdTomato+ cells (*R26R-tdTomato-flox/wt*, induced by *Krt5-rtTA;TetO-Cre* in the presence doxycycline) in the E18.5 mammary epithelium (magenta), together with volume rendering of isolated cells (gray). EpCAM staining (cyan, bottom right) was used for creating an epithelial surface rendering to mask mesenchymal and to highlight epithelial tdTomato expression (magenta, top right). **(B)** Quantification of tdTomato+ cells sphericity in the tip (at ≤100 µm distance from the landmark point in the tip) and duct (at >100 µm distance from the landmark point in the tip; *n*^tip^ = 27 cells, *n*^duct^ = 49 cells, from nine branches). **(C)** Visualization of Fucci2a cell tracks, color-coded for mean velocity (fire LUT, black→white = low→high), in an elongating mammary epithelial branch from a 3D time-lapse confocal video of an ex vivo cultured mammary explant (*K14-Cre;R26R-Fucci2a-flox/wt*). **(D)** Comparison of mean velocity of tracked cells between tip (defined as the region visibly enriched with M/G2/S cells) and duct (*n*^tip^ = 600 cells, *n*^duct^ = 1,814 cells, from nine elongating branches, four experiments). **(E)** Distribution of mean velocities of cell tracks with respect to their distance to the leading edge of the tip, where the trends of M/G2/S and G1/G0 tracks are shown as cyan and magenta lines, respectively (*n*^M/G2/S^ = 785, *n*^G1/G0^ = 1,629, from nine branches, four experiments). **(F)** Percentages of cell tracks per video in the tip and duct that switch from basal outermost cell layer (≤6 µm from the surface of the gland) to inner epithelial compartment (>6 µm from the surface of the gland) and vice versa (*n* = 9 branches, four experiments). **(G)** Tendency of inner cells in the tip and duct to move closer to the epithelial surface (calculated based on cell distance to the epithelial surface) in elongating branches (*n*^tip^ = 403 cells, *n*^duct^ = 1,212 cells; from nine branches, four experiments). **(H)** Percentages of cell tracks per video that start within the tip (M/G2/S-enriched region) and end in the duct and vice versa (*n* = 9 branches, four experiments). **(I)** We analyzed if cells at the end of their tracks were closer or further away from the leading edge of the tip than where they started (change in cell distance to the tip; positive values = closer, negative values further) and compared cells that started within the tip and duct (*n*^tip^ = 600 cells, *n*^duct^ = 1,814 cells, from nine branches, four experiments). **(J)** The change in cell-leading edge distance was plotted against the starting position of the tracks with the trends of M/G2/S and G1/G0 tracks shown as cyan and magenta lines, respectively (*n*^M/G2/S^ = 785 cells, *n*^G1/G0^ = 1,629, from nine branches, four experiments). **(K)** Visualization of Fucci2a cell tracks, color-coded according to mean velocity, before tip bifurcation (left), during tip widening (middle), and after cleft formation (right) from 3D time-lapse confocal videos of ex vivo cultured mammary explants (fire LUT, black→white = low→high). **(L)** Mean velocity of tracks derived from 10 bifurcating tips from seven experiments, divided in two groups based on whether they were closer to future tips (blue) or future cleft (red) and compared to those in the duct (gray) according to whether they started before bifurcation (*n*^tip^ = 272, *n*^cleft^ = 77, *n*^duct^ = 154), during tip widening (*n*^tip^ = 688, *n*^cleft^ = 279, *n*^duct^ = 1,152) or after the cleft became visible (*n*^tip^ = 1,098, *n*^cleft^ = 1,143, *n*^duct^ = 1,645). **(M)** We analyzed if cells at the end of their tracks were closer or further away from the leading edge/future cleft than where they started and compared cells that started within the parent tip and duct (boundary defined based on curvature of the neck) before bifurcation (*n*^tip^ = 346, *n*^duct^ = 174), during tip widening (*n*^tip^ = 879, *n*^duct^ = 1,240) and after the cleft first appeared (*n*^tip^ = 1,997, *n*^duct^ = 1,534). Correlation between variables in E and J was assessed with the Pearson coefficient (R) and P value for the linear correlation given. Data shown in D, G, I, L, and M represent the median (line) with 25th and 75th percentiles (hinges) plus 1.5× interquartile ranges (whiskers). Data shown in B, F, and H represents average ± SD. Statistical significance was assessed with the two-tailed *t* test (B–D and G), paired two-tailed *t* test (F and H), and Wilcoxon’s rank sum test (I, L, and M); *, P ≤ 0.05; **, P ≤ 0.01; ***, P ≤ 0.001; ****, P ≤ 0.0001.

**Figure S5.**
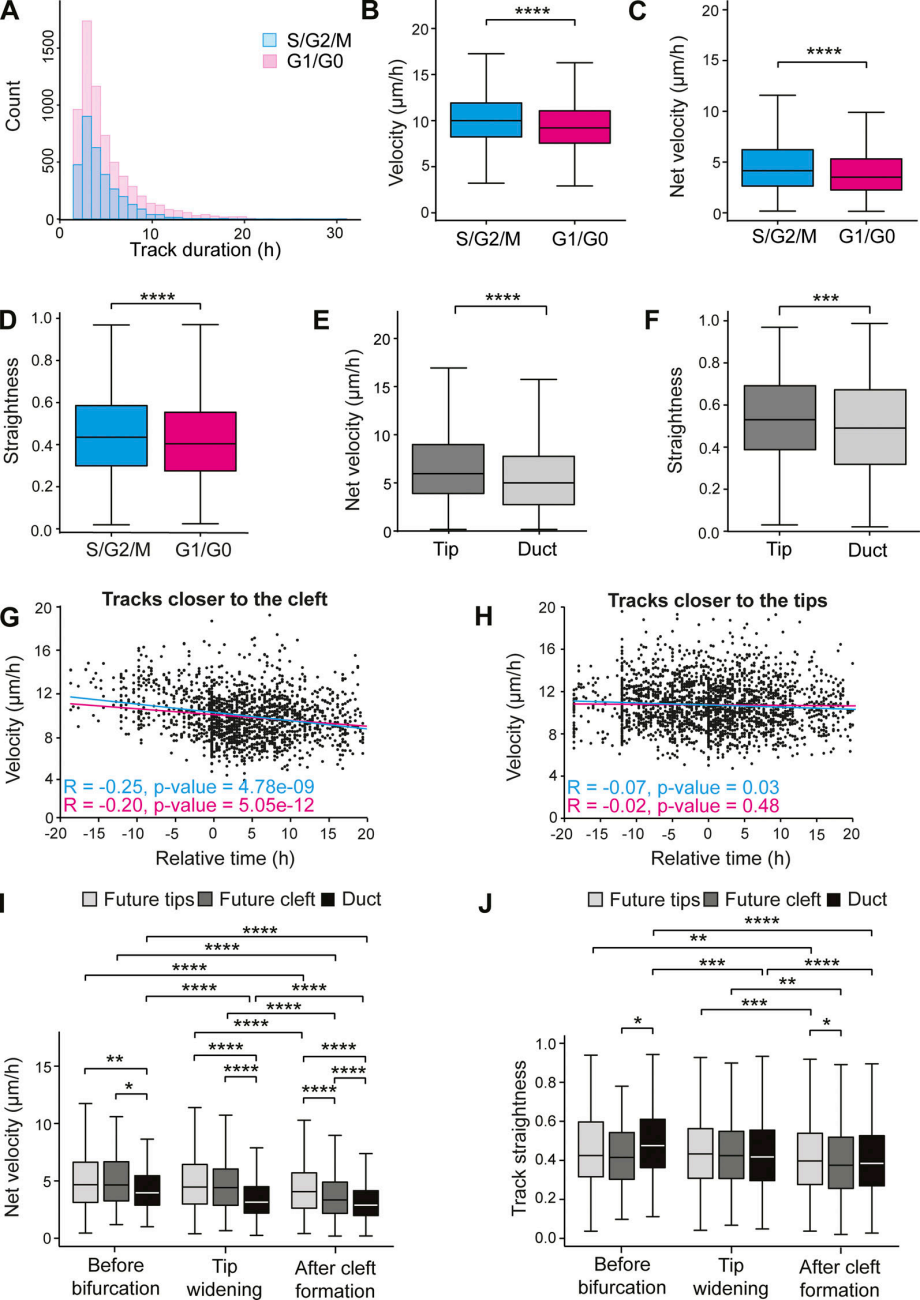
**Cell motility parameters based on cell cycle phase and track location. (A)** Histogram of cell tracks of different duration in all time-lapse imaging videos (*n*^S/G2/M^ = 3,191, *n*^G1/G0^ = 6,584 from seven experiments). **(B–D)** Comparison of the mean velocity (B), net velocity (C), and straightness (D) of tracks of S/G2/M and G1/G0 phase cells (*n*^S/G2/M^ = 3,191, *n*^G1/G0^ = 6,584, seven experiments). **(E and F)** Comparison of the net velocity (E) and straightness (F) of tracks within tip and duct regions (see schematics in Fig. 5 for how regions were defined) of nine elongating branches (*n*^tip^ = 600, *n*^duct^ = 1,814), four experiments. **(G and H)** Velocity of tracks within bifurcating tips that were closer to the future cleft (G) or closer to future tips (H) at different time points relative to the appearance of the cleft at T0 (*n*^tip^ = 2,058, *n*^cleft^ = 1,499 from 10 videos, seven experiments). **(I and J)** Net velocity (I) and straightness (J) of cell tracks within bifurcating tips, divided in those closer to the future cleft and those closer to the future daughter tips, compared those within the duct (boundary between parent tip and duct defined based on curvature of the neck), based on whether they started before bifurcation (*n*^tip^ = 272, *n*^cleft^ = 77, *n*^duct^ = 154), during tip widening (*n*^tip^ = 688, *n*^cleft^ = 279, *n*^duct^ = 1,152) or after cleft formation (*n*^tip^ = 1,098, *n*^cleft^ = 1,143, n^duct^ = 1,645). Correlation between variables in G and H was assessed with the Pearson coefficient (R) and P value for the linear correlation given. Data shown in B–F, I, and J represents the median (line) with 25th and 75th percentiles (hinges) plus 1.5× interquartile ranges (whiskers). Statistical significance was assessed with two-tailed *t* test (E and F) and Wilcoxon’s rank sum test (B–D, I, and J); *, P ≤ 0.05; **, P ≤ 0.01; ***, P ≤ 0.001; ****, P ≤ 0.0001.

To address whether branch elongation involves epithelial cell motility, we surmised that mammary cells would have a motile phenotype, characterized by an elongated rather than cubical epithelial cell shape. To analyze cell shapes in 3D, mammary epithelial cells were sparsely labeled with cytosolic tdTomato using a doxycycline inducible *K5-rtTA;TetO-Cre* and analyzed in fixed glands. We observed that most cells had a spindle-like shape with pointed edges (Fig. 5 A). Cells at the tip, closer to the leading front of elongation (≤100 µm), were less spherical than cells further away in the duct (Fig. 5 B). To study cell motility in elongating branches, we performed automated cell tracking based on nuclear Fucci2 expression in the live imaging dataset (Video 5). As the Fucci2a expression is temporarily lost during mitosis, we did not attempt to construct cell lineages by combining tracks based on different Fucci2a fluorophores. The length of the tracks generated was similar for both fluorophores, with a median track length of 3.7 h for Fucci2a-green and 4 h for Fucci2a-red cells (tracks shorter than 2 h were excluded; Fig. S5 A). Overall, cells in the M/G2/S phase of the cell cycle were slightly faster and more persistent compared to cells in G1/G0 phase of the cell cycle (Fig. S5, B–D). To assess if any differences exist in cells’ velocity along the elongating branches, we mapped all tracks based on their position with respect to the tip (Fig. 5 C). Cells in the tip, defined as the region with higher proportion of M/G2/S phase cells, were significantly faster and more persistent compared to the cells in the trailing duct (Fig. 5 D; and Fig. S5, E and F; and Video 5). The velocity of the tracks formed a descending gradient with increasing distance from the leading front of the tip (Fig. 5 E).

**Video 5.** **Elongating branch followed by time-lapse confocal microscopy in an ex vivo cultured mammary gland expressing Fucci2a dual nuclear cell cycle reporter (M/G2/S = cyan, G1/G0 = magenta).** 3D images were collected every 20 min for a total of 10 h (related to Fig. 5 C, display rate = 3 frames/s). Tracking of Fucci2a fluorescent nuclei is shown with maximum intensity projection (MIP) and with velocities indicated by color.

Next, we explored how differential cell motility affects cells’ contribution to different tissue domains. First, we investigated if cell movement contributes to epithelial surface expansion and analyzed the movement of cells between basal and inner “compartments.” We observed a trend where the basal cells moved more often to the inner compartment than vice versa, regardless of their position along the branch—tip or duct (Fig. 5 F). To assess if there was any overall trend of radial movement of inner cells, we analyzed the position of cells relative to the epithelial surface and found that overall, their positions were maintained (Fig. 5 G). The gradient of cell velocities in the elongating branch (Fig. 5 E) indicated that slower tip cells may get left behind and end up in the trailing duct. To investigate this possibility, we analyzed the destination of the tip and duct cells and found that significantly more tip cells ended up in the duct than duct cells in the tip (Fig. 5 H). To evaluate cell lag along the elongating branch, we measured the distance of cells to the leading edge of the tip at the start and end of the track: cells in the trailing duct lagged behind significantly more than cells in the tip (Fig. 5 I). The farther away from the leading edge the cell was, the greater the lag (Fig. 5 J).

As the front/center part of the tip transforms into the branch point once the tip bifurcates, we hypothesized that cell movement may change at this location. Therefore, we examined cells’ velocity in branches undergoing terminal bifurcation (Fig. 5 K and Video 6). Remarkably, once the cleft had been established, cells at the branch point appeared to slow down. To confirm this observation, we analyzed tracks in the parent tip based on their proximity to landmarks positioned at the future daughter tips and the emerging cleft and stratified the tracks based on when they were generated—during branch elongation, tip widening, or cleft formation. Tracks that were closer to the future cleft became slower, while the cells closer to the daughter tips remained motile (Fig. 5 L and Fig. S5, G–J). Once the cleft had been established, the branch point cells were significantly slower than the cells of the daughter tips, suggestive of their localized immobilization. We hypothesized that immobilization of the cells at the branch point may restrict the flow of lagging cells to the trailing duct, preventing its further elongation. Indeed, we found that the flow of cells from the tip toward the trailing duct was significantly reduced during tip bifurcation (Fig. 5 M).

**Video 6.** **Bifurcating branch followed by time-lapse confocal microscopy in an ex vivo cultured mammary gland expressing Fucci2a dual nuclear cell cycle reporter (M/G2/S = cyan, G1/G0 = magenta).** 3D images were collected every 20 min for a total of 26 h (related to Fig. 5 K, display rate = 3 frames/s). Tracking of Fucci2a fluorescent nuclei is shown with maximum intensity projection (MIP) and with velocities indicated by color.

In conclusion, analysis of cell behaviors in elongating branches revealed a gradient of cell movement along the elongating branch. This creates a retrograde flow of cells that contributes to the elongation of the trailing duct. Once a branch point is generated, the flow of the cells is interrupted, possibly due to immobilization of the branch point cells.

### The invasion of the tip is facilitated by directional cell movement, which is redirected during tip bifurcation

**Figure 6.**
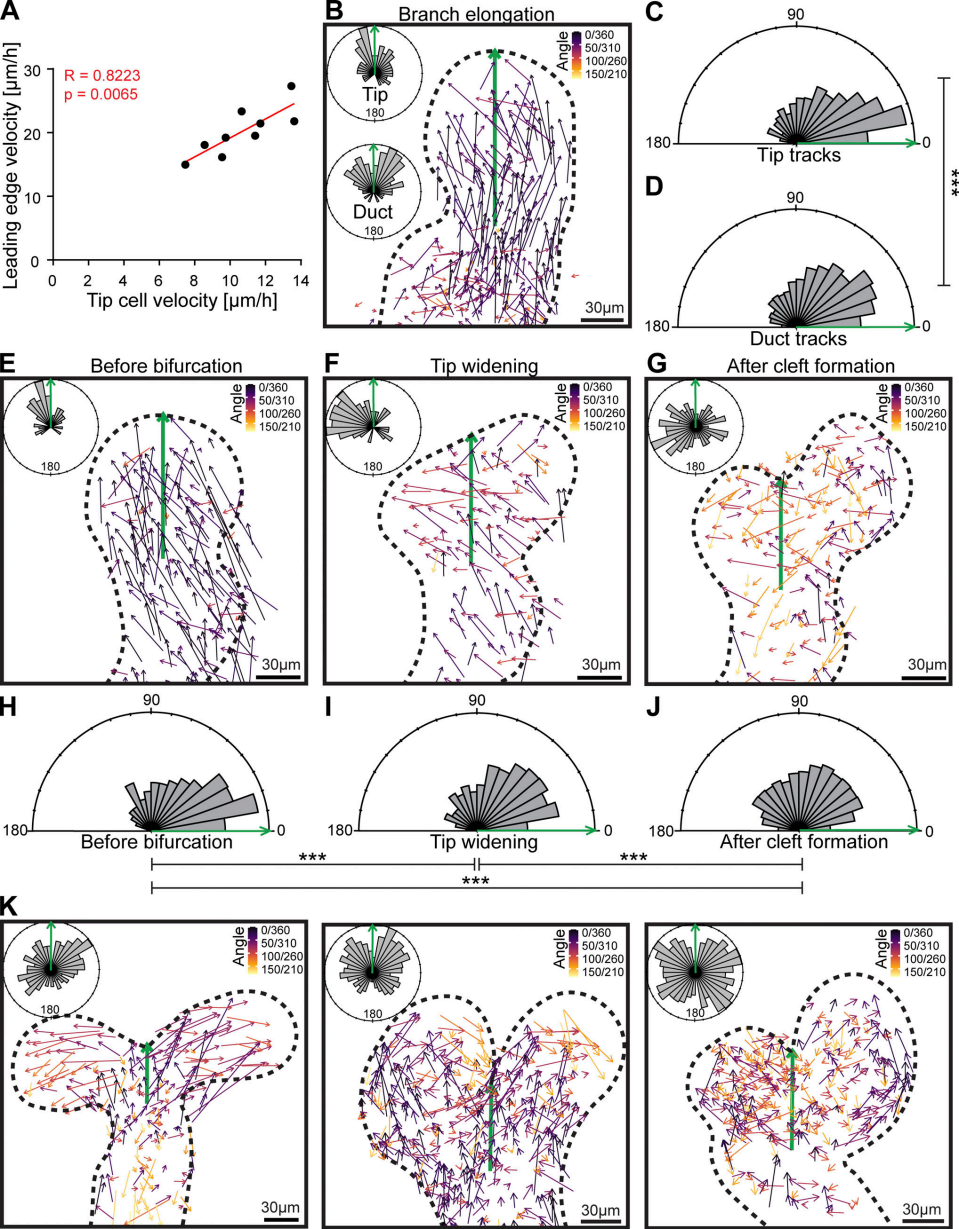
**Invasion of the tip is facilitated by directional cell movement, which is redirected during tip bifurcation. (A)** Correlation of leading edge velocities of elongating branches with the average velocity of tip cell tracks (*n* = 9, four experiments). **(B)** Cell displacement vectors color-coded based on their angle (inferno LUT, black = 0/360°, yellow = 180°) to the displacement vector of the leading edge of the elongating branch (green). Parallel cell movement corresponds to angles closer to 0/360 degrees, while antiparallel movement correspond to angles closer to 180 degrees. In addition, Rose diagrams are shown to further illustrate left- and right-sided angles in the 360° range for tracks within the tip and duct. **(C and D)** From the aggregated data of nine different elongating branches (four experiments), we merged left- and right-sided angles to the range of 0–180 degrees (parallel movement = 0°, antiparallel movement = 180°) and show rose diagrams of the angles of tip cell displacement vectors (contained within the area where M/G2/S cells were visibly enriched; C) and duct cell displacement vectors (D) against the displacement vector of the leading edge as a reference (*n*^tip^ = 600, *n*^duct^ = 1,814). **(E–G)** Cell displacement vectors that were created before bifurcation (E), during tip widening (F), and after cleft formation (G), color-coded based on their angle (inferno LUT, black = 0/360°, yellow = 180°) to the reference vector (green) running from the center of the neck to the leading edge/future cleft (green arrow). To assess the degree of asymmetric cell movement, we also show rose diagrams that distinguish between left- and right-sided angles of the displacement vectors in the 360° range for tracks within the bifurcating tip (boundary defined based on curvature of the neck). **(H–J)** From the aggregated data of 10 different bifurcations (seven experiments), we merged left- and right-sided angles to the range of 0–180 degrees and show rose diagrams of the angles of tip cell displacement vectors relative to the reference vector, depending on whether they started and ended before bifurcation (*n* = 214; H), during tip widening (*n* = 475; I) and after the cleft first became visible (*n* = 2,214; J). **(K)** Examples of cell displacement vectors from different bifurcations during cleft formation, showing variable patterns of directional cell movement with rose diagrams to distinguish between left- and right-sided angles in the 360° range (inferno LUT, black = 0/360°, yellow = 180°) within the bifurcating tips (boundary defined based on neck curvature). Correlation between variables in A was assessed with the Pearson coefficient (R) and P value for the linear correlation given. Uniformity of angles was tested with the Rayleigh test and statistical significance between groups was assessed with the Watson's Two-Sample Test of Homogeneity; *, P ≤ 0.05; **, P ≤ 0.01; ***, P ≤ 0.001; ****, P ≤ 0.0001.

Branch elongation in the mammary gland involves the forward advancement of the tip into the surrounding stromal tissue. We found that velocity of tip correlated with the velocity of tip cells (Fig. 6 A), implying tips may be propelled by cell movement. To investigate if the direction of cell movement is consistent with the direction of the tip, we analyzed the vectors of cell displacement in elongating branches (Fig. 6 B). We measured the angle of the cell displacement vectors to the vector of tip displacement and found that the bulk of the cells moved to the same direction as the tip (Fig. 6, C and D). Furthermore, tip cells displayed significantly smaller angles than the cells in the trailing duct (Fig. 6, C and D), suggesting that their movement was more coherent. These results indicate that the direction of the invading tip may be governed by directionality of the tip cells.

Upon tip bifurcation, two daughter tips are generated that begin to elongate branches in two different directions. To explore how this is reflected at the cellular level, we visualized cell displacement vectors in bifurcating tips (Fig. 6, E–G). The direction of the cells gradually became more dispersed, which correlated with the emergence of the daughter tips. We compared the displacement vectors of the cells to a reference vector, running from the center of the neck to the future cleft, and calculated the angle in between. We found that before widening of the tip, cells were moving parallel to reference vector (Fig. 6 H), much like in the elongating branches (Fig. 6, B–D). However, once the tip begun to widen and the cleft became visible, the angles became significantly wider, indicating that cells moved more perpendicular to the parental tip axis (Fig. 6, I and J). Examining cell displacement vectors in different bifurcating tips revealed two separate streams of perpendicular cell movement entering into the daughter tips (Fig. 6 K).

In conclusion, these results suggest that invasion of the tip is mediated by directional cell migration, which becomes redirected between the two daughter tips during tip bifurcation.

### Cell movement is an active migratory process rather than cell displacement caused by proliferation

**Figure 7.**
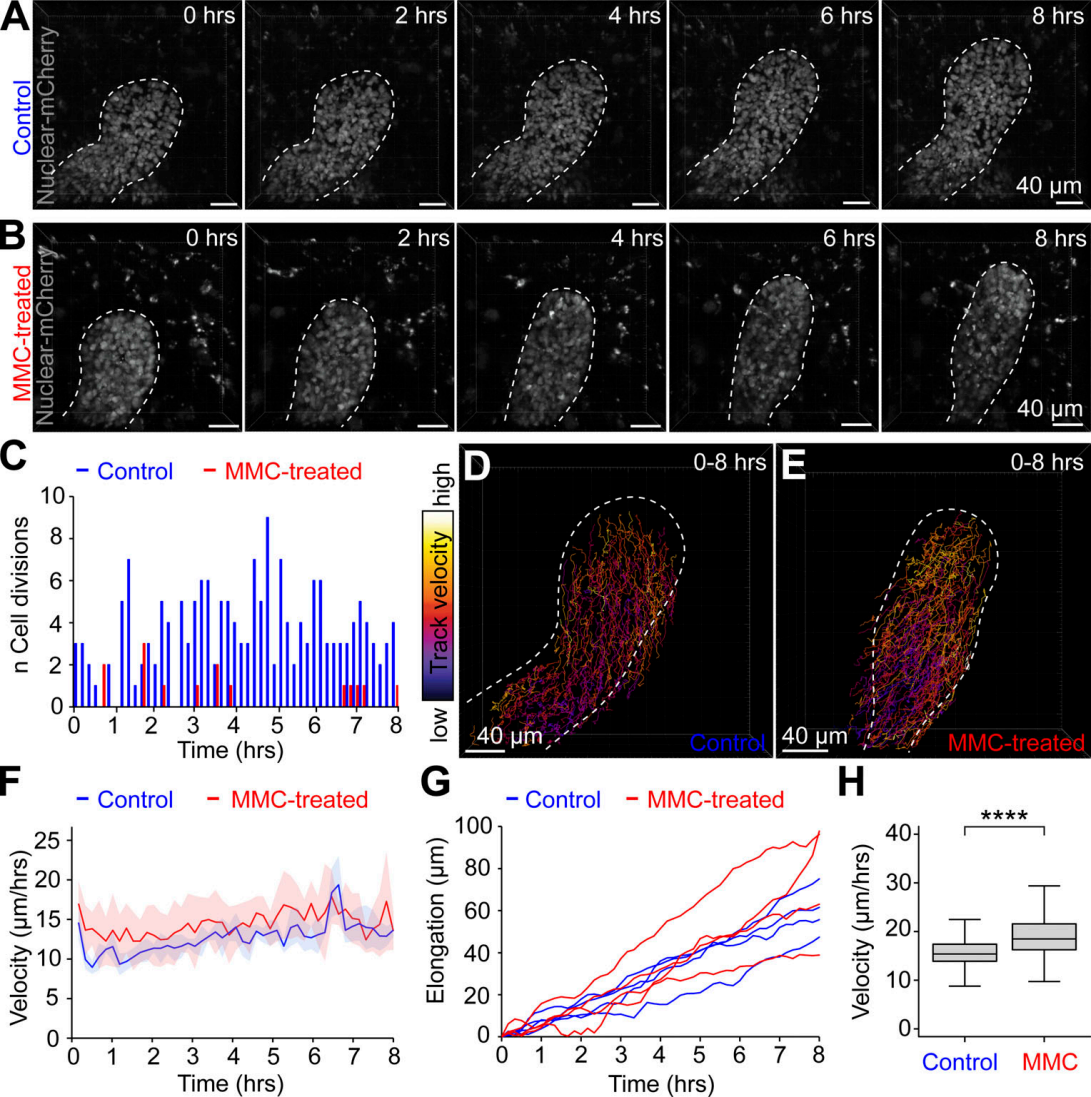
**Cell movement is an active migratory process rather than displacement propelled by proliferation. (A and B)** Representative captions of an 8-h time-lapse live imaging sequence depicting the elongation of branches expressing nuclear mCherry in the epithelium (*K14-Cre*;*R26R-RG*) after 2-h treatment with vehicle (A) or with 1 µg/ml of MMC (B). **(C)** A histogram enumerating the cell divisions detected in MMC-treated and control branches (*n* = four branches per condition of equal length on average, imaged in three experiments). **(D and E)** The tracks of cells generated between 0 and 8 h after treatment with vehicle (D) or MMC (E) where the mean velocity of the track is color-coded (fire LUT, black→white = low→high). **(F)** Cells were pooled within 100 µm of the leading edge and the velocity of tracked cells between frames per tip plotted as a function of time (*n* = four branches per condition from three experiments). Data shown represent average (line) ± SD (shaded region). **(G)** The leading edge of each tip was tracked frame by frame and the distance to the starting point plotted as a function of time. **(H)** Tracks within 100 µm of the leading edge were compared between control and MMC-treated branches (*n*^control^ = 539, *n*^MMC^ = 395, four branches each from three experiments). Data shown represent the median (line) with 25th and 75th percentiles (hinges) plus 1.5× interquartile ranges (whiskers). Statistical significance was assessed with the Wilcoxon rank sum test; ****, P ≤ 0.0001.

Our results so far indicate that branch elongation is not directly powered by cell proliferation (Fig. 3) but facilitated by cell motility (Figs. 5 and 6). However, to establish that cell movement is not in essence the displacement of cells caused by cell divisions, we investigated the motility of cells under cell cycle inhibition by live imaging. Mammary explants expressing a constitutive nuclear fluorescent reporter (*K14-Cre;R26R-RG*), were treated with MMC or a vehicle control for 2 h and imaged 8 h immediately after the treatment in a confocal setup (Fig. 7, A and B). The efficacy of the treatment was confirmed by scoring of cell division events, visible based on nuclear mCherry expression. In total, only 15 cell divisions were detected in MMC-treated explants as opposed to the 179 of control branches (Fig. 7 C). Tracking of cells indicated that MMC did not inhibit cell motility over the 8 h imaging period (Fig. 7, D–F), which is consistent with elongation of the branches analyzed (Fig. 7 G). On the contrary, cell movement was significantly faster under cell cycle inhibition (Fig. 7 H), implying that cell divisions may restrain cell movement.

### Tip bifurcation involves narrowing of the cleft and can be initiated independently of the mesenchyme

**Figure 8.**
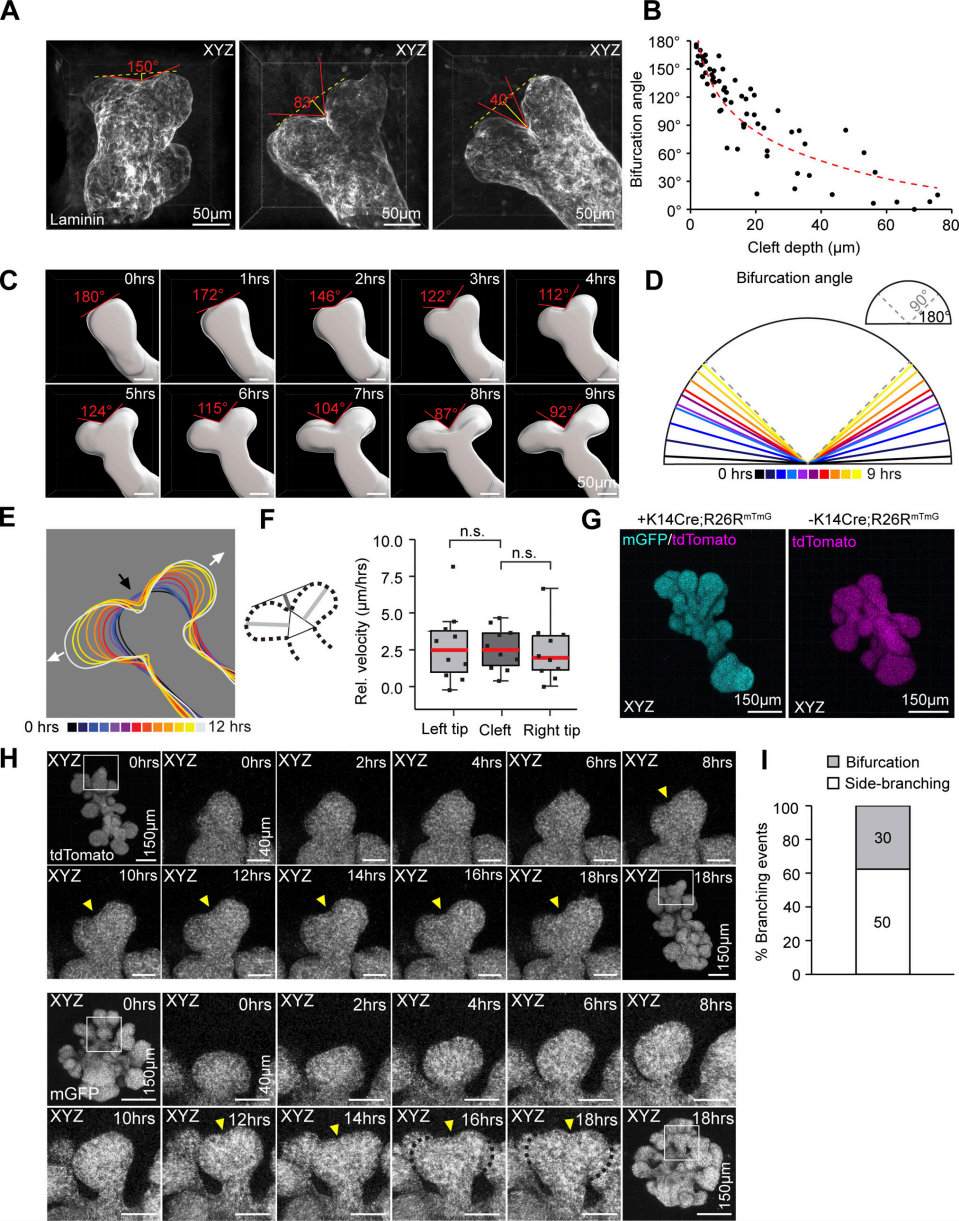
**Tip bifurcation involves narrowing of the cleft and can be initiated independent of the mesenchyme. (A)** Maximum intensity projections of bifurcating tips, imaged from E17.5–E18.5 mammary glands, with a shallow cleft (left) and deeper clefts (middle, right) visible based on laminin-staining. **(B)** The angle and depth of the cleft was measured as indicated in A and plotted for 65 bifurcations from 30 glands of 13 embryos. **(C)** Images of an epithelial surface rendering of a bifurcating tip from a time-lapse imaging video of an epithelial Fucci2a expressing ex vivo cultured mammary gland, at 2-h intervals following the appearance of the cleft with the bifurcation angle indicated. **(D)** Average bifurcation angles from 10 Fucci2a videos (seven experiments) at incremental hours starting from the appearance of the cleft as indicated by color. **(E)** Outlines of the epithelial surface rendering of a bifurcating tip at incremental hours from the start of the time-lapse video as indicated by color (ingression of the cleft is indicated by a black arrow and advancement of the daughter tips by white arrows). **(F)** The depth of the cleft and the length of the daughter tips were measured from time-lapse imaging videos of surface rendered bifurcating tips after the appearance the cleft and their relative velocity calculated based on duration of the video (*n* = 10, 7 experiments). **(G)** Maximum intensity projections of mammary epithelial rudiments, derived from *R26R-mTmG* embryos with (left) or without (right) *K14-Cre*. **(H)** Maximum intensity projections of an epithelial rudiment cultured and imaged in 3D Matrigel for a period of 18 h. The boxed region follows a bifurcating tip at 2-h intervals. **(I)** Quantification of branching events from 26 rudiments of four time-lapse imaging experiments. Data shown in F represents the median (line) with 25th and 75th percentiles (hinges) plus 1.5× interquartile ranges (whiskers). Statistical significance was assessed with the paired two-tailed *t* test.

Tip bifurcation involves establishment of a cleft between the two daughter tips that becomes a permanent part of the branch point. In other organs, the cleft has been described either as a localized obstruction or a wedge that splits the tip (Sakai et al., 2003; Kim et al., 2015). To visualize clefts in vivo, we performed whole mount 3D imaging of the basement membrane visualized with the laminin antibody (Fig. 8 A). Measurements of the angle and depth of the cleft showed that shallow (i.e., nascent) clefts were associated with wider angles and deeper clefts with narrower angles (Fig. 8 B). To monitor clefting in real time, we used epithelial cell surface rendering to measure cleft angles in bifurcating tips and found that the cleft was initially wide and became narrower over time (Fig. 8, C and D), suggesting that clefting in the mammary gland is a process of gradual narrowing. To investigate to what extent cleft formation contributes to tip bifurcation, we analyzed movement of the cleft and daughter tips. Visualization of the epithelial outline revealed that both advancement of the daughter tips and ingression of the cleft contributed to the shaping of the tip (Fig. 8, E and F). The outward movement of the daughter tips and the inward movement of the cleft was almost equal, suggestive of their mutual contribution (Fig. 8 F).

Whether tip bifurcation can be accomplished chiefly by epithelial cells, or whether the action of mesenchymal cells is necessary, is unclear. Classical tissue recombination experiments suggest that the mesenchyme determines the mode of branching events (Kratochwil, 1969). Next, we investigated whether tip bifurcations are possible in the absence of the mesenchymal tissue. To this end, E16.5 sprouting organoids were cultured for 2 d (Fig. 8 G) and then imaged once per half hour to an hour by 3D confocal microscopy for a minimum of 19 h (Fig. 8 H). Although patterning of the epithelium was clearly atypical, with shorter and more densely packed branches, distinct tip splitting and budding events could be discerned. Quantification revealed that about 40% of the branching events were tip splitting events (Fig. 8 I and Video 7) indicating that some aspects of tip bifurcation ability are intrinsic to the epithelium.

**Video 7.** **Mesenchyme-free sprouting organoids expressing mGFP or tdTomato (derived from *R26R-mT/mG* embryos with or without K14-Cre expression) were imaged with time-lapse confocal microscopy for a minimum of 19 h with 30–60 min intervals (related to** Fig. 8 H**).** Maximum intensity projections of four 3D organoids are shown in the top row with bifurcation events outlined by red boxes and magnified on the bottom row. Display rate = 2–3 frames/s.

### Tip bifurcation involves patterning of ERK-signaling activity

**Figure 9.**
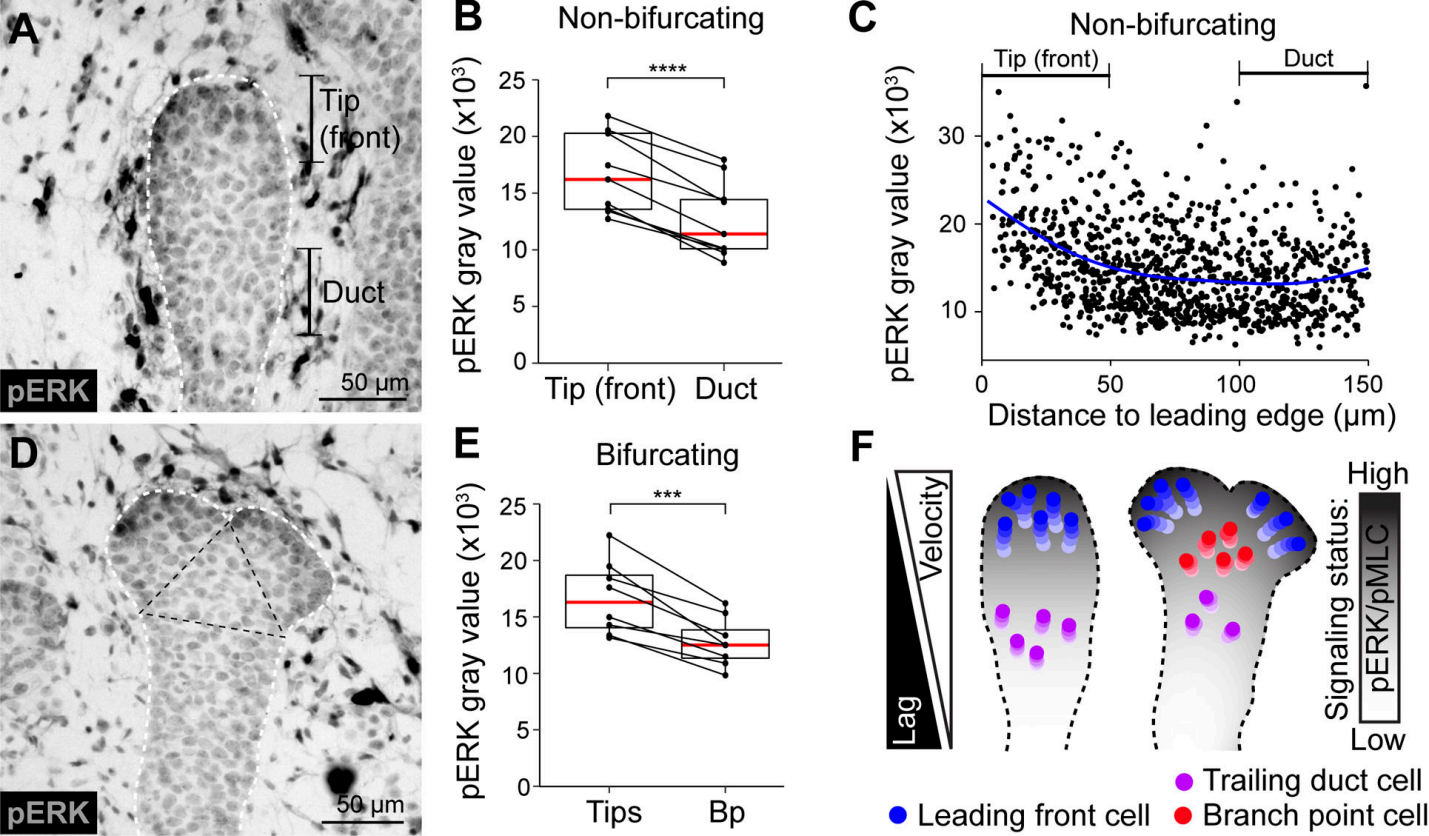
**ERK signaling is active at the leading fronts of tips. (A)** pERK in a non-bifurcating branch (epithelial outline demarcated by white dotted line) detected by chromogenic antibody staining in a paraffin section of an E18.5 mammary gland. **(B)** Quantitation of nuclear pERK intensity within 0–50 µm (tip front) and 100–150 µm (trailing duct) range of distances of the leading edge (*n* = 10 branches from two embryos). **(C)** Distribution of pERK intensities per cell against cell distance to the leading edge (*n* = 10 branches from two embryos). **(D)** pERK in a bifurcating branch (epithelial border marked by white dotted line and branch point marked by black dotted line) detected by chromogenic antibody staining in a paraffin section of an E18.5 mammary gland. **(E)** Quantification of nuclear pERK intensity between the daughter tips and the branch point (*n* = 9 bifurcating branches of two embryos). **(F)** An illustration depicting the signaling domains that were identified and the cell behaviors that were associated with these domains in the present study (blue cells = leading front cells, red cells = branch point cells, purple cells = trailing duct cells). Data shown in B and E represents the median (line) with 25th and 75th percentiles (hinges) plus 1.5× interquartile ranges (whiskers) with individual paired data-points illustrated by connecting lines. Statistical significance was assessed with the two-tailed paired *t* test; ***, P ≤ 0.001; ****, P ≤ 0.0001.

As tip bifurcation involves localized changes in cell motility and proliferation, we investigated whether these behaviors might be accompanied by changes in signaling status. Epithelial receptor tyrosine kinase (RTK) signaling plays a conserved role across multiple organs as a positive regulator of branching morphogenesis (Wang et al., 2017). RTK signaling is partially relayed through the MAPK (mitogen-activated protein kinase)/ERK cascade. We assessed the levels of ERK activity by staining of phosphorylated ERK1/2 in tissue sections of E18.5 mammary glands. The signal was found in the nucleus in the mammary epithelium, concentrated toward the leading front of the tip (Fig. 9 A)—the same domain where we had previously identified pMLC to be enriched (Fig. 4 E). This domain (0–50 µm from the leading edge) contained significantly more pERK activity compared to a segment of equivalent length behind the tip (100–150 µm from the leading edge; Fig. 9 B), and overall, there was a steep gradient of elevated signaling toward the leading edge (Fig. 9 C). In bifurcating tips, pERK was found at the newly defined leading fronts of the daughter tips (Fig. 9 D), significantly enriched relative to the branch point region in between (Fig. 9, D and E), again mirroring the signaling domains of pMLC (Fig. 4, F and G). These results suggest that ERK signaling is patterned in the epithelium, and the signaling domains are found at the leading front, where cell motility and contractility are heightened (Fig. 9 F).

## Discussion

Branching morphogenesis creates an arborized epithelium by iterating branch elongation and branch point generation in growing tips of all branched organs. How these two actions are executed and integrated in the mammary gland has not been established mainly due to lack of comprehensive real-time evidence. Here we have studied branching morphogenesis of the embryonic mammary gland within the native inductive mesenchyme by ex vivo live imaging. We asked how cell cycle activity and cell motility, two basic elements of epithelial construction, are coordinated within bifurcating terminal branches.

The branching tips of several mammalian organs are enriched in proliferative cells (Varner and Nelson, 2014), but whether differential growth makes branches grow longer or bifurcate, has not been confirmed so far. The TEBs of the pubertal mammary gland are comprised of cycling stem cells, whereas cells in ducts are more differentiated (Scheele et al., 2017). Our examination of embryonic mammary glands revealed that similar to the pubertal glands (Cannata et al., 2010; Giraddi et al., 2015; Scheele et al., 2017), tips were more proliferative than the ducts, though proliferation in embryonic ducts appears to be higher than in pubertal ducts. Despite higher proliferation, cells do not seem to accumulate in the tip, as tip width remained unchanged during branch elongation ex vivo and only slightly increased with branch length in vivo. We found that cells get displaced from the tip based on their differential motility. Although ductal elongation is apparently fueled by a reservoir of proliferative tip cells, examining the effects of global cell cycle repression let us to conclude that elongation of the duct cannot be based on spatially controlled cell divisions, asymmetric or otherwise. Inhibitor experiments in stroma-free mammary epithelial organoids also point toward this direction (Huebner et al., 2016). Importantly, we further show that cell movement in elongating branches is independent of cell cycle activity and cell divisions may even limit the rate of cell movement during elongation.

Elongation of embryonic mammary epithelial branches was coupled with directional cell migration, gradually increasing toward the leading edge of the tip. In the vertebrate vasculature and *Drosophila* trachea, branches elongate upon the invasive migration of “leader cells” occupying the pointed edges of tips (Varner and Nelson, 2014). According to our data, a substantial proportion of basal tip cells rearranged into the inner compartment, suggesting that the leading-edge position may be more transient. Dynamic movements have also been reported in organoids and within TEBs in vivo (Ewald et al., 2008; Scheele et al., 2017; Dawson et al., 2021; Lloyd-Lewis et al., 2022). Stratification seems to be a necessary event for collective cell migration to occur (Ma et al., 2022), consistent with the observed stratification of embryonic branch tips and TEBs. Thus, branch elongation can be considered to be a process of propulsion, where epithelial cells leverage each other to push the TEB forward (Goodwin and Nelson, 2020). We propose that branch elongation is supported by differential cell motility along the branch, coupled with directional cell competition to the leading-edge position. This creates a retrograde flow of lagging cells from tip to duct, consistent with clonal lineage tracing data in pubertal glands (Scheele et al., 2017). Notably, we failed to find evidence of radial cell intercalation, which contributes to branch elongation in organoids (Neumann et al., 2018; Pfannenstein and Macara, 2023 *Preprint*). Perhaps such intercalations are not necessary in embryonic branches, as in contrast to TEBs, the diameter of growing tips is not substantially larger than that of the trailing duct, except just prior to bifurcation. Additionally, the epithelium is fully stratified, unlike in TEBs, where cells organize around a central lumen and transition from multi-layered to a ductal bi-layer.

Very little is known of branch point formation in the mammary gland. We now find that it is associated with cell cycle repression and reduced cell motility, in contrast to the behaviors that we observe in the tips of elongating branches. Tip proliferation was not acutely required for new branches to be initiated. Bifurcations however were unstable, and the daughter tips became reabsorbed by the parent tip in the absence of proliferation—perhaps the bifurcated geometry is more challenging to maintain with limited cell numbers while branch points restrict cell movement. Whether, on the other hand, cell cycle repression is required at the branch point remains an open question, but it is reasonable to assume that branch points cease to grow once established. In other organs, bifurcations are not known to be associated with local differences in cell cycle activity (Lang et al., 2021). In the salivary gland, differential expansion of the inner and outer bud epithelium creates a mechanical instability, which causes the more expansive surface cell sheet to buckle (Wang et al., 2021; Kim et al., 2021). Surface expansion involved subsurface cell division and reinsertion of new surface cells. We did observe a trend of basal-to-inner cell movement in elongating and bifurcating branches alike but could not assess whether these cells divided or returned to the surface. Overall, inner-to-basal cell movements were rare and thus unlikely to contribute to a buckling mechanism. Lineage tracing data indicate that in the pubertal gland, basal and luminal compartments are maintained separately (Van Keymeulen et al., 2011; Davis et al., 2016; Wuidart et al., 2016). Those basal cap cells that do venture into the TEB body undergo apoptosis (Paine et al., 2016; Dawson et al., 2021).

One key aspect in branch point generation is the establishment of a cleft that ultimately changes the geometry of the tip. While salivary cleft appears as a narrow space between two cells (Kadoya and Yamashina et al., 2010), in the embryonic mammary gland, clefts first appeared as shallow indentations that became narrower as they deepened over time similar to lung bifurcations (Schnatwinker and Niswander, 2013; Kim et al., 2015). Proliferation and outward motility of cells of the daughter tips is likely to be part of the shape change. Intriguingly, pMLC localized to the leading front of tips, suggesting that NMII activity may contribute to tip outgrowth/elongation. In cultured salivary glands, a similar enrichment in the outer-bud region has been observed (Kim et al., 2021). A recent examination of lungs also identified pMLC in association with epithelia with a positive curvature (Hirashima and Matsuda, 2022 *Preprint*). NMII inhibition abrogated branch point generation in ex vivo cultured mammary glands and also in mesenchyme-free sprouting organoid cultures, indicating that contractile actin is required for epithelial branching. We interrogated potential NMII activators and confirmed a role for the RhoA/ROCK pathway, which can be induced by various cell surface receptors, including RTKs and adhesion receptors (Mosaddeghzadeh and Ahmadian, 2021). Actomyosin contractility is known to power multiple cell behaviors, including cell motility that, according to the present study, contributes not only to branch elongation but also tip bifurcation.

We found that cell motility contributes to bifurcations in two ways: while cell immobilization helps to establish branch points, directional cell movement contributes to elongation of the daughter tips. Changes in cell motility were spatiotemporally coordinated with changes in cell cycle activity, implying that they may be controlled by a localized inductive/repressive cue. The conditions that might trigger such a cue however are enigmatic, given the variation in branch point frequency in the mammary gland (Lindström et al., 2022 *Preprint*). It may involve local modulation of RTK signaling activity, which is a universal regulator of branching morphogenesis (Wang et al., 2017). In the mammary gland, RTK signaling is likely activated by Fgf (fibroblast growth factor) ligands (Lu et al., 2008; Parsa et al., 2008), which has been associated with tip cell motility and proliferation (Lu et al., 2008; Huebner et al., 2016; Neumann et al., 2018). Indeed, we found that ERK signaling, one of the downstream drivers of RTK pathway, was activated at the leading fronts of tips and suppressed at branch points. Kidney bifurcations utilize RTK signaling via RET (ret proto-oncogene) receptor activation (Ihermann-Hella et al., 2014), which drives cell sorting movements to the daughter tips (Riccio et al., 2016). Branch point generation in the lung has been modeled based on a ligand–receptor-based Turing type of mechanism, involving tissue-restricted expression of the Fgf10–Fgfr2b (fibroblast growth factor 10–fibroblast growth factor receptor 2b) ligand–receptor pair (Lang et al., 2021). Downstream of Fgf signaling, a negative feedback loop between lung epithelial curvature and ERK activation mediates epithelial folding through the actin cytoskeleton (Hirashima and Matsuda, 2022 *Preprint*). Although a stratified epithelium may not fold the same way, the interaction between ERK signaling and the contractile actin cytoskeleton at the leading front might be highly relevant (Fig. 9 F).

In conclusion, our results suggest that directional cell movement advances the tip of a branch, while differential cell motility and proliferation feeds cells from the tip into the trailing duct during its elongation. Bifurcation of the tip involved local attenuation of both cell proliferation and movement at the branch point while establishment of the daughter tips was facilitated by their sustained proliferation and directional “outward” cell movement. In a simplified view, our findings imply that branching morphogenesis might be accomplished by modulating the same set of cell behaviors in branch tips to alternate between elongation and branch point generation. Tip bifurcations initiated also in mesenchyme-free cultures, suggesting that they are at least to some extent, intrinsic to the epithelium. While we were revising our manuscript, tip bifurcations in mesenchyme-free organoids and suppression of cell motility in the cleft area of bifurcating tips were reported (Neumann et al., 2023). However, stromal collagen deposition is likely to contribute to cleft stabilization and establishment of the bifurcation angle in vivo (Nerger et al., 2021). Future imaging studies are awaited to decipher how pathways with recognized roles in mammary gland development such as Wnt, Tgfβ1 (transforming growth factor β1), Bmp (bone morphogenic protein), and Eda/NF-κB (ectodysplasin A/nuclear factor κ light chain enhancer of activated B cells; Spina and Cowin, 2021) regulate the critical cell behaviors identified in this study.

## Materials and methods

### Ethics statement

All mouse experiments were approved by local ethics committee and the National Animal Experiment Board of Finland (licenses KEK19-019, KEK22-014, and ESAVI/2363/04.10.07/2017). Mice were euthanized with CO_2_ followed by cervical dislocation.

### Mouse lines

The conditional *R26R-RG* (Shioi et al., 2011) and *R26R-Fucci2a-flox/flox* (Mort et al., 2014) reporter mice were maintained in C57BL/6 background. The STOP cassette surrounded by loxP sites was removed with ubiquitous *PGK-Cre* (Lallemand et al., 1998) to generate the constitutive *R26R-Fucci2a-del/del* reporter mice, also maintained in C57BL/6 background (Lan et al., 2023 *Preprint*). *R26R-Fucci2a-del/del* mice were crossed with wild-type NMRI mice for cell cycle analysis from fixed samples. *R26R-mTmG* mice obtained from The Jackson Laboratory (Stock 007576) were in ICR background. *R26R-tdTomato-flox/flox* mice, also from The Jackson Laboratory (Stock 007914), were maintained in C57BL/6 background. *K14-Cre43* mice (Andl et al., 2004; hereafter *K14-Cre*) in NMRI background were used for epithelial expression of conditional fluorescent reporters, except for *R26R-RG*, which was crossed with another *K14-Cre* line (Huelsken et al., 2001) with a more sparse expression. Mice carrying both *K5-rtTA* (Diamond et al., 2000) and *TetO-Cre* (Mucenski et al., 2003) transgenes (*K5-rtTA;TetO-Cre*) in NMRI background were used for doxycycline-inducible sparse labeling of epithelial cells. For sparse labeling, *R26R-tdTomato-flox/flox* females were mated with *K5-rtTA;TetO-Cre* bi-transgenic males. On the 13th day from the plug (E13.5), females were injected with doxycycline hyclate (D9891-5G; Sigma-Aldrich) dissolved in sterile PBS, in concentration of 4 µg per 1 g of mouse body mass. Mice were kept in 12 h light–dark cycles, and food and water were available ad libitum.

### Whole-mount staining and imaging

For whole-mount confocal microscopy of fixed samples, E17.5–E18.5 female embryos, or 5–6 d cultured mammary explants were used. After removing the head and limbs from E17.5–E18.5 embryos, a longitudinal incision was made along the midline from the neck toward the groin, and two flanks of skin with five pairs of mammary glands were peeled to the sides and cut off. The flanks were first spread on a plastic surface and then submerged and fixed with 4% PFA in PBS overnight at +4°C on an orbital shaker, followed by three washes with PBS. Mammary explants were fixed for 1 h at room temperature and washed thrice. For immunofluorescence staining, samples were blocked and permeabilized with 5% goat or donkey serum, 0.3% Triton X-100 in PBS for 1 h at room temperature. Primary antibodies were prepared in blocking/permeabilization buffer and incubated with samples for 2–3 d at +4°C. For EpCAM staining, rat anti-mouse CD326 was used as a 1/1,000 dilution (552370; BD Biosciences). Rabbit anti-Laminin was used as a 1/1,000 dilution (L9393; Sigma-Aldrich). After washing the samples four times for 1–2 h in PBS, secondary antibodies and 5 µg/ml of Hoechst (H3570; ThermoFisher Scientific) were added in blocking/permeabilization buffer and incubated with the samples for 2–3 d at +4°C. Secondary antibodies conjugated to Alexa 488, 568, and 647 were purchased from Thermo Fisher Scientific and used as a 1/500 dilution. For staining keratins, 1% Triton X-100 was used during blocking and antibody incubation steps. Both anti-cytokeratin 14 (K14; RB-9020-P; Thermo Fisher Scientific) and anti-cytokeratin 8 (K8; TROMA-I; DSHB) antibodies were used at a 1/500 dilution. For staining of actin filaments, 10 U/ml of Alexa Fluor 568 Phalloidin (A12380; Thermo Fisher Scientific) was added to the secondary antibody mixture. Before mounting, samples were washed as described above.

Stained flanks with Hoechst were placed under the Zeiss Lumar V12 microscope equipped with Apolumar S 1.2× objective, epidermis facing down, and mammary glands visualized under the UV light. Individual glands were carefully dissected out together with the surrounding mesenchyme and mounted on slides using Vectashield (Vector Laboratories). To preserve the 3D structure, several layers of sticky tape were used as a spacer between the slide and the coverslip. Mammary glands were imaged at room temperature using Leica DM6 or DMI8 point-scanning confocal microscopes, both equipped with HC PL APO 20×/0.75 IMM CORR CS2 objective and operated with the Las X software. For cell cycle analysis, 3D image stacks were acquired of the entire mammary gland number 3 using tile imaging with 0.2 µm/px xy resolution and 1–2 µm z step size. Mammary gland whole-mount staining of keratins were imaged at ∼0.35 µm/px xy resolution and at a 2 µm z step size. For closer analysis of terminal branches/tips, images were acquired from mammary glands 1–5 using 0.1 µm/px xy resolution and 1 µm z step size.

### Immunostaining and imaging of paraffin sections

Samples were collected for sectioning from E18.5 female embryos as described above for whole-mounts, fixed overnight at +4°C, washed with PBS, processed into paraffin and embedded such that the flanks of skin lay flat at the bottom of the mold. Serial sections of 5 µm thickness were cut and sections examined carefully under a dark field microscope to identify the bifurcating and non-bifurcating tips of branches. Slides were deparaffinized, followed by antigen retrieval in TE-buffer (20 mM Tris-HCl, pH 9; 1 mM EDTA) with 0.5% Tween in antigen-unmasking device (Aptum Biologics Ltd). Staining of pERK from sections with the rabbit pERK1/2 (1/300, 4370; Cell Signaling Technology) antibody was performed as previously described (Ihermann-Hella et al., 2014) using the BrightVision poly HRP-Anti-Rabbit IgG secondary antibody (DPR110HRP; Immunologic) and DAB peroxidase substrate kit (SK-4100, Vector) for detection. pERK-stainings were imaged in Shandon Immu-Mount at room temperature with Leica DM6000B upright fluorescence widefield microscope operated on Las X software and using a 20X/0.7N.A. HCX PL APO objective and Leica DMC2900 CMOS color camera for capturing images. For immunofluorescence staining with mouse anti-p63 (1/400 dilution, ab735; Abcam), mouse anti-pMLC (1/300 dilution, 3675S; Cell Signaling Technology), and rat anti-K8 (1/400 dilution) antibodies, sections were treated 5 min with 3% H_2_O_2_ in PBS, followed by blocking with 10% donkey serum in PBS-0.1%-Tween20 for 1 h at room temperature. To block endogenous mouse IgGs, slides were incubated with the M.O.M blocking reagent (MKB-2213-1, Vector Laboratories) for 1 h at room temperature. After washing three times 5 min with PBS-0.1%-Tween, sections were incubated with primary antibodies overnight at +4°C in PBS-0.1%-Tween20. Incubation with secondary antibodies (Thermo Fisher Scientific) was performed 1.5–2 h at room temperature. Immunofluorescence stainings were imaged in Shandon Immu-Mount with the Leica DMI8 point-scanning confocal microscope operated on Las X software and using a 40X/1.25N.A. HC PL APO objective.

### Ex vivo culture of embryonic mammary glands

Ex vivo culture of embryonic mammary glands was performed according to a previously established protocol (Lan et al., 2022). Left and right flanks of abdominal-thoracic skin containing five pairs of mammary buds were dissected out from E13.5 embryos. The tissues were treated for 20–40 min at +4°C with 1.25 U/ml of Dispase II (4942078001; Sigma-Aldrich) in PBS and 3–4 min at room temperature with a pancreatin-trypsin mixture (2.5 mg/ml pancreatin [P3292; Sigma-Aldrich] and 22.5 mg/ml trypsin in Thyrode’s solution, pH 7.4). The enzyme solution was replaced with culture media (10% FBS in 1:1 DMEM/F12 supplemented with 100 µg/ml ascorbic acid, 10 U/ml penicillin and 10 mg/ml streptomycin) and tissues rested on ice for a minimum of 30 min. The epidermal layer was pulled away with small needles, leaving the mesenchymal tissue with the mammary buds. The tissues were collected on square pieces of Nuclepore polycarbonate filter (WHA110605; Whatman) and suspended on metal grids on a 3.5 cm Ø plastic dish, filled from below with culture medium. The explants were cultured up to 7 d in +37°C with air and 5% CO_2_, replacing the media every other day. Blebbistatin (20 µM concentration, B0560; Sigma-Aldrich), Y27632 (50 µm concentration, 10005583; Cayman Chemicals), or ML-7 (20 µm concentration, 11801; Cayman Chemicals) were added into the culture media on day 5–6 of culture and left for 24 h. MMC was added into the culture media as a 1 µg/ml concentration, left for 2 h, and replaced with normal culture media, where explants were cultured for 22 h. Branching was assessed in explants derived from *K14-Cre*;*R26R-mT/mG* embryos by quantifying the number of tips/gland from images taken before treatment and after culture at room temperature using the Zeiss Lumar V12 Stereoscope operated with Zen Blue software and equipped with Apolumar S 1.2× objective and Axiocam MRm camera. EdU labeling was performed by treating the explants with 10 µM EdU in culture media for 1 h at +37°C with 5% CO2 in air atmosphere. Staining of EdU-labeled samples was performed with the Click-iT EdU Cell Proliferation Kit for Imaging (C10340; Thermo Fisher Scientific) according to the manufacturer’s instructions, with the following modifications: explants were fixed for 1 h with 4% PFA in PBS at room temperature, permeabilized with 0.2% Triton X-100 in PBS for 30 min and then blocked for 30 min with 3% BSA in PBS. Incubation in Click-iT reaction cocktail was 2 h. Stained explants were imaged as described for whole mounts.

### Mesenchyme-free mammary rudiment culture

Mammary epithelial rudiments were cultured in 3D Matrigel as previously described (Lan et al., 2022). Skin flanks with mammary rudiments 1–5 were collected from E16.5 female embryos as described above for later stages. Tissues were treated for 1 h at +4°C with 2.5 U/ml of Dispase II in PBS, followed by 5–7 min of treatment with pancreatin-trypsin at room temperature. The tissues were then rested on ice for 30–60 min in culture medium. The epidermis was pulled away with needles and mammary rudiments 1–3 liberated from the mesenchyme—at this stage the thoracic mammary rudiments have developed 2–3 small branches. The mesenchymal tissue was cleaned away with needles and the buds were washed by pipetting through a 10 μl tip several times. The epithelial rudiments were transferred onto the bottom of a 3.5-cm Ø dish with 10 μl of culture media. The media was removed and replaced with a 10 μl drop of growth-factor reduced Matrigel (356231; Corning), 3–5 mammary rudiments/drop. The rudiments were dispersed as not to touch each other or the bottom of the dish. The Matrigel was allowed to solidify at +37°C and when solidified, 2 ml of organoid culture media (1:1 DMEM/F12 supplemented with 10 U/ml penicillin, 10 000 µg/ml streptomycin, 1X ITS Liquid Media Supplement [I3146; Sigma-Aldrich] and 2.5 nM hFGF2 [CF0291; Sigma-Aldrich]). Mammary rudiments were cultured up to 3 d at +37°C with 5% CO_2_ in air atmosphere, with the medium replaced on day 2. Blebbistatin was added on day 2 as a 20 µM concentration. Brightfield images were taken with the Zeiss Lumar V12 Stereoscope operated with Zen Blue software and equipped with Apolumar S 1.2× objective and Axiocam MRm camera. Edu labeling and imaging of stained organoids was performed as described for explants and whole mounts.

### Live imaging

#### Wide-field imaging of branching morphogenesis ex vivo

To monitor the effects of MMC on branching morphogenesis by live imaging, mammary explants were dissected from E13.5 *K14-Cre*;*R26R-mT/mG* embryos and prepared for ex vivo culture as described above, but directly placed with a Pasteur pipette to the apical side of a 24 mm Ø Trans-well insert to be cultured on a 6-well plate (Falcon). Tissue culture medium was added up to the level of the insert (1–1.5 ml) and explants cultured for 3 d in a tissue culture incubator at 37°C, 5%CO_2_. Imaging was performed using an inverted 3I Marianas microscope with an environmental chamber (+37°C air with 6% CO_2_) and a long working distance air objective (10×/0.30 N.A. EC Plan-Neofluar Ph1 186 WD = 5.2 M27). A LED light source 183 (CoolLED pE2 with 490 nm/550 nm) was used for exposure, and widefield images were acquired with the Andor Neo sCMOS camera operated with Slidebook software at multiple positions at 3-h intervals over a period of 93 h between 3 and 7 d of culture. Between 45 and 47 h, samples were treated with 1 µg/ml MMC or with the same volume of PBS in fresh normal culture media. After the treatment, media was replaced and imaging continued under normal conditions.

#### Confocal imaging of mammary epithelial cell behaviors ex vivo

Mammary explants from E13.5 *K14-Cre;R26R-Fucci2a-flox/wt* or *K14-Cre;R26R-RG-flox/wt* embryos were established as described above and first cultured 5–6 d in standard culture conditions. Explants were then carefully transferred on Nucleopore filters to the membrane bottom of a 5 cm Ø Lumox dish (94.6077.410; Sarstedt) with the tissue side facing down. The edges of the Nucleopore filter were rapidly attached to the Lumox membrane by touching with fine-tipped forceps dipped in Vetbond tissue adhesive (0200742529; 3M Science), taking care not to touch the explant at the center. A 50-μl drop of culture media was added on top of each explant to prevent the tissues from drying. After attaching all the explants, 4–5 ml of culture media was added onto the dish. Imaging was performed with Leica DMI8 inverted laser scanning microscope with an environmental chamber, operated with Las X software. To assess the effects of cell cycle inhibition on cell movement, a four-chambered flexiPERM disc (Sarstedt) was placed on the dish with 1–2 explants in each well and cultured with or without 1 µg/ml MMC for 2 h in the microscope environmental chamber before collecting images. The dish was placed on a 3D printed sample holder fashioned for the Lumox dish that can be inserted to the frame of a 6-well plate sample holder on the motorized stage. The environmental chamber was set to +37°C air with 5% CO_2_ and 90% humidity, and the samples were equilibrated there for 1 h before imaging. HC PL APO 20×/0.75 CS2 air objective was used for time-lapse imaging on 4–6 positions at 20 min intervals for up to 40 h (for the Fucci2a reporter) or at 10 min intervals (for the RG reporter). 3D image stacks were acquired at each position with a 0.5–0.8 µm/px xy resolution and 2 µm z step size, using 600 Hz bi-directional scanning. The pinhole was opened to 2AU to reduce the laser exposure needed. For analysis, videos were cropped and time points limited to include only terminal branches that elongated or bifurcated (10–28 h). Samples treated with or without MMC were imaged for 8 h.

#### Imaging of epithelial sprouting organoids in mesenchyme-free culture

3–5 E16.5 epithelial mammary rudiments, embedded in 10 μl drops of Matrigel, were applied onto 3.5cm Ø glass bottom dishes. The sprouting organoids were prepared from *R26R-mT/mG* embryos with or without *K14-Cre* and cultured for 2 d before imaging. Organoids were imaged in +37°C air with 5% CO_2_ at multiple positions with an inverted Leica DMI8 microscope operated with Las X software using the HC PL APO 10×/0.40 CS2 air objective at 1-h intervals over a period of 19–23 h. Images stacks were acquired with 1–2 µm/px xy resolution using 5–7 µm z steps.

### Fixed image analysis

#### Analysis of whole glands

To analyze E18.5 mammary glands, a surface rendering of the epithelium was first generated in Imaris (Bitplane) using the hand drawing tool on every 3–5 slices. A binary mask was created and exported to Fiji. To measure the width of tips, the line tool was used on the maximum intensity projection of the masked epithelial rendering. In cases of overlap, the mask was cleared in part before the projection was made to reveal the tip to be measured. For cell cycle analysis, the surface rendering was used for masking the Fucci2a signal in the mesenchyme so that epithelial expression could be better visualized. Quantification of Fucci2a expressing cells was performed using the Spots function in Imaris software with 6 and 7 µm spot size for G1/G0 and M/G2/S cells respectively. The spots were made slightly smaller than the measured diameter of the nuclei to ensure recognition neighboring cells that with a larger spot size would have been recognized as one cell. The intensity threshold was manually set for each image to ensure the overlay of spots on the fluorescent nuclei. A binary mask was generated based on the G1/G0 and M/G2/S spots. The mask was used as a template to generate the same number of smaller spots that were spatially more separated. This was necessary to avoid merging of neighboring spots while the dimensions of the image were modified in xy to yield cubic voxels. A mask was generated based on the smaller spots and for the epithelial surface rendering. The masked channels were exported to Fiji and the spots were fed to the 3D Objects counter and loaded to the 3D manager of 3D ImageJ Suite (Ollion et al., 2013) as separate regions of interest. The distance of the spots to the surface of the gland was determined by mapping them onto a 3D chamfer distance map, created with Morpholib-J from the masked epithelial rendering (Legland et al., 2016). The data was stratified into outer (≤6 µm) and inner cells (>6 µm). Marker images were created using the point tool to mark the position of tips, ducts, and branch points—for ducts, the marker was placed squarely between two branch points or between the tip and the last branch point. 3D geodesic distance maps were generated running inside the epithelial rendering from each marker to map the distances of spots. Spots were pooled at a given distance from each marker to calculate the % M/G2/S cells. For tips, cells were pooled at a distance of ≤100 µm. For ducts and branch points, the distance varied based on the thickness of the branch, between 50 and 100 µm. The length of the terminal branches was determined based on measuring the distance of the latest branch point to the tip, using a 3D geodesic distance map generated using markers placed in tips.

For analysis of K14 and K8 expression in mammary epithelial cells, 3D image stacks of keratin and Hoechst stainings were pre-processed using the ImageJ plugin, Noise2Void (https://ieeexplore.ieee.org/document/9098336) with the N2V train and predict module to improve the signal. The training was performed with 200 epochs, 200 steps per epoch, a batch size per step of 64, and a patch shape of 64. The neighborhood radius was adjusted to either 3 or 5 based on the quality of the images. Cells were detected based on Hoechst staining in TrackMate (Ershov et al., 2022) of Fiji using a spot size larger than the nuclei itself. To verify that the spots could be used to probe the levels of surrounding keratin expression within the cells, they were filtered based on K14 and K8 intensity and the overlay of spots with positive cells confirmed manually. The intensity values of keratins were then derived from all the spots and the distances of spots to the gland surface were mapped based on a distance transformation, created from an epithelial surface rendering. The cells that appeared to be negative for both keratins (gray value <25) were filtered out from the analysis).

#### Analysis of terminal branches with bifurcating and non-bifurcating tips

Bifurcating tips were analyzed more closely from separate image stacks, where a surface rendering of the epithelium was created either based on EpCAM or laminin staining. The angle of the cleft was triangulated in Imaris using the measurement tool. A binary mask was created based on the surface rendering and another one marking the position of the cleft. The binary channels were exported to Fiji and a maximum intensity projection was created. A straight line was drawn from the front of one daughter tip to the other and the distance of this line to the cleft marker was measured, signifying the depth of the cleft. To analyze cell cycle distribution in the bifurcating tip in Imaris, the surface rendering was cut by the neck and at the root of each daughter tip to create separate volumes for daughter tips and the branch point where the percentage of M/G2/S could be calculated. To quantify the intensity of phalloidin staining, the surface rendering was used for separating epithelial and mesenchymal phalloidin staining into separate channels. Spots were generated to mark the position of the cleft and the daughter tips as well as the neck at both sides. A distance transformation was created emanating from each spot and used as basis for a spherical volume around it of a given distance. The sum of intensities in the epithelium and mesenchyme inside the volume was derived. The number of the corresponding pixels was calculated based on the mask of the epithelial rendering and used for determining mean intensities. For analysis of pERK staining intensity in sections, images were converted to 16-bit gray scale and the histograms of all images were normalized for equal saturation (0.02%). As all epithelial cells seemed to display a basal level of pERK activity, we used it as a basis for nuclear segmentation. The images were inverted in Fiji, and segmentation was carried out with the StarDist plugin for 2D images (Schmidt et al., 2018). Small (<20 µm) and large regions (>120 µm) were filtered out to include only single nuclei for deriving pERK intensities. The location of the cells was inferred from an epithelial mask that was generated with the free-hand tool and in bifurcating tips, a separate triangular masked region was generated for the cleft using the polygon selection tool (as shown in Fig. 9 D). In non-bifurcating tips, the distance of the nuclei to the leading front, demarcated by a point selection, was determined based on a distance transformation. For analysis of pMLC intensity on sections, the average gray value was compared between regions of interest containing the leading front (0–50 µm) and the immediate ductal region behind a non-bifurcating tip (100–150 µm) or between the daughter tips and a branch point of a bifurcating tip.

#### Cell shape determination

Samples were collected from doxycycline-induced *Krt5-rtTA;TetO-Cre;R26R-tdTomato-flox/wt* female embryos at E18.5 and whole-mount staining and imaging was performed as described above. For cell shape analysis, 3D image stacks were acquired of mammary epithelial branches with 0.15 µm/px xy resolution and 1.04 µm z step size. For image analysis, a surface rendering of the epithelium was generated in Imaris. Surfaces were created based on tdTomato fluorescence using a manually set threshold according to the intensity levels and surface grain size set to 0.3 µm. The position of the cells was determined by relative distance to manually placed landmark. For the tip, cells were pooled at a fixed distance of ≤100 µm from the landmark, while cells located at a distance >100 µm were considered as part of the duct. Statistical output of surface rendering was used to analyze cell sphericity.

### Live video analysis

#### Quantification of branching events and branch elongation rates

The time-lapse videos in TIFF format were opened in Fiji and time series registered with the StackReg plugin (Thévenaz et al., 1998). Branching events were quantified and identified by carefully inspecting the adjacent time points. The length of branches was measured frame to frame using the Segmented Line tool in Fiji.

#### Morphometric analysis of tips

The Fucci2a time-lapse videos were drift corrected in Imaris against the origin of the gland, which is marked by a cluster of intensely fluorescent G1/G0 cells. A surface is created around this cluster and tracking of its center is used for drift correction. The field of view was cropped to facilitate faster analysis of terminal branches. The videos were shortened, when necessary, to focus on analyzing terminal branches exclusively when they elongated. For bifurcating tips, the videos were normalized on the time scale based on the timepoint where the cleft first became apparent. The G1/G0 and M/G2/S channels were merged in Imaris and used as a basis to trace a surface rendering by hand at every 2–3 slices, carefully including all epithelial cells. For morphometric analysis, a mask of the intact epithelial rendering was created and exported to Fiji. The width of the tips was measured with the line tool frame by frame. For bifurcating tips, the length of the tip was also measured from the center of the neck to the cleft and further up to a straight line drawn from one daughter tip to the other—the depth of the cleft being the difference between these two measurements. The angle of the cleft was measured with the Angle tool by tracing along the edges of the cleft toward the daughter tips. The surface rendering was also cut into separate volumes to demarcate different tissue domains. In elongating branches, there was no clear curvature to exactly mark the neck of the tip. Therefore, the tip and the trailing duct were separated by cutting the surface rendering where the M/G2/S cells appeared to be enriched. In bifurcating tips, the curvature of the neck became apparent and could be used as a cutting site. The daughter tips and the branch point could be separated into volumes by cutting diagonally from the cleft towards the neck of the tip.

#### Analysis of Fucci2a spots and tracks

6 and 7 µm spots were created in Imaris for G1/G0 and M/G2/S cells, respectively. A distance transformation was created from the intact epithelial surface rendering to determine their distances from the surface of the gland. The % of M/G2/S was calculated inside each volume described above. For cell tracking, the diameter of M/G2/S and G1/G0 nuclei was measured and 8 and 9.3 µm spots were created for G1/G0 and M/G2/S cells, respectively. The maximum distance that a spot may move from one frame to the next was restricted to 8 and 9.3 µm and no gaps were tolerated to ensure that tracking followed the same nuclei. The fidelity of tracking was confirmed by manually tracking a subset of cells. The position of tracks was determined by their mean distance to manually placed tissue landmarks or to the surface of the gland based on distance transformations. The front of the tip in an elongating branch was demarcated by a spot and the distances of tracks referred to it. In bifurcating tips, the position of tracks was determined based on whether they were closer to landmarks placed in daughter tips or the cleft. When indicated, the position of the cells at the start and end of the track were compared either to investigate if they had moved from one volume to another or whether they were closer or further away from a tissue landmark. Parameters of cell movement were compared between differently positioned tracks (Velocity = Track length/Track duration, Net velocity = Track displacement length/Track duration, Straightness = Track displacement length/Track length). In bifurcating tips, tracks were also compared between different stages of morphogenesis, defined based on morphological measurements. The directionality of the cells was analyzed based on their coordinates at the start and end position of the track as displacement vectors. The directionality was determined as the angle between the displacement vector and a reference vector. For the elongating branch, the displacement vector of the tissue landmark placed at the leading front of the tip served as the reference. For the bifurcating tip, the reference vector was the line drawn from the midpoint of the neck towards the cleft.

#### Tracking of R26R-RG cells

4D image stacks were pre-processed with Noise2Void as described above to improve visualization/detection of the nuclear reporter. For this purpose, the XYZT images were converted to XYZ format and after processing transformed back to XYZT for further analysis. To analyze cell velocities, nuclei were automatically detected and tracked in Imaris using 6 µm spotsize and 8 µm steps (no gaps allowed and tracks with a duration <2 h were excluded). Cell divisions were carefully screened by eye based on the characteristic appearance of nuclei: cells were judged to divide if they first appeared in anaphase and then in telophase/cytokinesis in the following frame.

### Statistics and data representation

For clarity, box plots were used to display variations within larger groups of data. When data contained <50 observations per group, individual data points were also shown.

Each group of data was tested with the Shapiro–Wilk test to estimate whether it was normally distributed. A Levene test was performed to establish whether variations of groups to be compared were equal. In cases where variations were not equal, a Welch *t* test was used instead of Student’s *t* test. If data was not normally distributed, a Wilcoxon’s signed rank test (paired) or Wilcoxon’s rank sum test (not paired) was performed. For pairwise comparison of multiple groups, a Bonferroni correction was used in calculating the P values. Correlation between variables was assessed with Pearson’s correlation coefficient and when appeared to be linear, was fitted to a linear model and a P value for the fit is provided.

### Online supplemental material

Fig. S1 shows distribution of cell cycle indicators and cell fate markers in E18.5 mammary glands. Fig. S2 shows characterization and live imaging of ex vivo cultured embryonic mammary glands. Fig. S3 presents branching events in MMC-treated and non-treated mammary explants. Fig. S4 shows perturbation of NMII activity in mammary explants and mesenchyme-free sprouting organoids. Fig. S5 shows analysis of tracking of Fucci2a fluorescent nuclei in elongating and bifurcating branches. Video 1 shows live imaging of Fucci2a cell cycle indicators during branch elongation. Video 2 shows live imaging of Fucci2a cell cycle indicators during tip bifurcation. Video 3 shows long-term live imaging of embryonic mammary gland branching morphogenesis. Video 4 shows long-term live imaging of embryonic mammary gland branching morphogenesis under MMC treatment. Video 5 shows tracking of Fucci2a cell cycle indicators during branch elongation. Video 6 shows tracking of Fucci2a cell cycle indicators during tip bifurcation. Video 7 shows tip bifurcations in mesenchyme-free sprouting organoids.
